# Systems-level analysis of NalD mutation, a recurrent driver of rapid drug resistance in acute *Pseudomonas aeruginosa* infection

**DOI:** 10.1371/journal.pcbi.1007562

**Published:** 2019-12-20

**Authors:** Jinyuan Yan, Henri Estanbouli, Chen Liao, Wook Kim, Jonathan M. Monk, Rayees Rahman, Mini Kamboj, Bernhard O. Palsson, Weigang Qiu, Joao B. Xavier

**Affiliations:** 1 Program for Computational and Systems Biology, Memorial Sloan-Kettering Cancer Center, New York, New York, United States of America; 2 Department of Biological Sciences, Duquesne University, Pittsburgh, Pennsylvania, United States of America; 3 Department of Bioengineering, University of California San Diego, La Jolla, California, United States of America; 4 Icahn School of Medicine at Mount Sinai, New York, New York, United States of America; 5 Infection Control, Department of Medicine, Memorial Sloan-Kettering Cancer Center, New York, New York, New York, United States of America; 6 Department of Biological Sciences, Hunter College & Graduate Center, CUNY, New York, New York, United States of America; EMBL-Heidelberg, GERMANY

## Abstract

*Pseudomonas aeruginosa*, a main cause of human infection, can gain resistance to the antibiotic aztreonam through a mutation in NalD, a transcriptional repressor of cellular efflux. Here we combine computational analysis of clinical isolates, transcriptomics, metabolic modeling and experimental validation to find a strong association between NalD mutations and resistance to aztreonam—as well as resistance to other antibiotics—across *P*. *aeruginosa* isolated from different patients. A detailed analysis of one patient’s timeline shows how this mutation can emerge *in vivo* and drive rapid evolution of resistance while the patient received cancer treatment, a bone marrow transplantation, and antibiotics up to the point of causing the patient’s death. Transcriptomics analysis confirmed the primary mechanism of NalD action—a loss-of-function mutation that caused constitutive overexpression of the MexAB-OprM efflux system—which lead to aztreonam resistance but, surprisingly, had no fitness cost in the absence of the antibiotic. We constrained a genome-scale metabolic model using the transcriptomics data to investigate changes beyond the primary mechanism of resistance, including adaptations in major metabolic pathways and membrane transport concurrent with aztreonam resistance, which may explain the lack of a fitness cost. We propose that metabolic adaptations may allow resistance mutations to endure in the absence of antibiotics and could be targeted by future therapies against antibiotic resistant pathogens.

## Author summary

Our incomplete understanding of complex molecular networks, the collateral effects of resistance mutations, and unknown functions in bacterial genomes hinders our ability to prevent and treat antibiotic-resistant infections. Here we combined clinical, experimental and computational approaches to investigate how a recurring loss-of-function mutation in the transcriptional regulator NalD of *Pseudomonas aeruginosa* can confer antibiotic resistance in patients acutely infected by this opportunistic pathogen, driving the rapid evolution of resistance. Computational modeling revealed metabolic pathways that could be involved in the pathogen’s response to the antibiotic resistance mutation, and which may help offset a fitness cost in the absence of antibiotics. These results provide insights on a recurrent driver of antibiotic resistance in a major human pathogen.

## Introduction

The rise of antibiotic resistant bacteria is a major global problem [1,2]. Predicting, preventing and treating antibiotic resistant infections present challenges that are best addressed with multidisciplinary approaches combining evolutionary, molecular and computational biology [3]. Bacteria can acquire resistance through horizontal gene transfer, but they can also repurpose mechanisms they already possess. Chromosomal point mutations, in particular, enable rapid rewiring of bacterial regulatory networks [4] and provide means to evolve antibiotic resistance rapidly—a major risk for patients receiving therapy.

*P*. *aeruginosa* is a Gram-negative pathogen and a main cause of hospital-acquired infections [5]. This pathogen is often studied in the context of chronic lung infections of cystic fibrosis patients where infections can last decades; during that time patients receive frequent and aggressive treatments that select for antibiotic resistance [6] and biofilm formation [7]. On the other hand, *P*. *aeruginosa* causes acute infections in immune-compromised patients where everything happens quicker [8,9]. Acute *P*. *aeruginosa* infection of cancer patients receiving immuno-suppressive therapy for bone marrow transplantation, for example, has the highest 7-day mortality rate among all infections that afflict these patients [10]. The mechanisms driving evolution of *P*. *aeruginosa* resistance *in vivo* in acutely-infected patients—even as they receive treatment—has arguably received less attention.

Aztreonam, a monobactam derivative of beta-lactams with low susceptibility to beta-lactamases, is an important defense against Gram-negative bacteria including *P*. *aeruginosa* [11]. Its use in cystic fibrosis patients started in 2010 and has increased steadily since then [12]. But recent work has shown that *P*. *aeruginosa* can acquire rapid resistance against aztreonam *in vitro*, often through chromosomal mutations in one of 19 genes linked to overexpression of efflux systems or on the cellular target of aztreonam [13]. These mutations reportedly decreased *in vitro* growth rates in the absence of antibiotics, indicating an associated fitness cost. Similar chromosomal mutations were also found in isolates from cystic fibrosis, which highlights their clinical relevance for the treatment of *P*. *aeruginosa* chronic infections [13]. Could the same type of chromosomal mutations drive a rapid evolution of antibiotic resistance in acutely infected patients, even as they receive antibiotic treatment? And if so, what system-level changes enable the pathogen to thrive?

Here we present a comparative analysis across dozens of clinical isolates to show that NalD—a transcriptional repressor of the MexAB-OprM efflux system—has the strongest association with aztreonam resistance in isolates from acutely infected patients. Then, we dissect the case of one particular acutely-infected patient in whom aztreonam resistance evolved *in vivo* during aztreonam therapy. We demonstrate that the resistance was acquired due to a loss-of-function mutation in NalD and caused overexpression of the MexAB-OprM efflux pump consistent with the known mechanism of resistance. However, we found no fitness cost in the aztreonam-resistant strain in the absence of the drug comparing to its closest susceptible isolate. By integrating a genome-scale metabolic model with transcriptomics data, we explored whether the resistant strain has developed metabolic adaptations to compensate for the resistance. The model revealed system-level changes beyond the primary mechanism of resistance that included adaptations in major metabolic pathways, which may explain the lack of a fitness cost. We discuss how understanding the metabolic adaptations that offset the fitness cost of resistance may pave the way to future therapies against antibiotic resistant infections.

## Results

### Aztreonam resistance is associated with NalD mutation in acutely-infected patients

**Fig 1:**
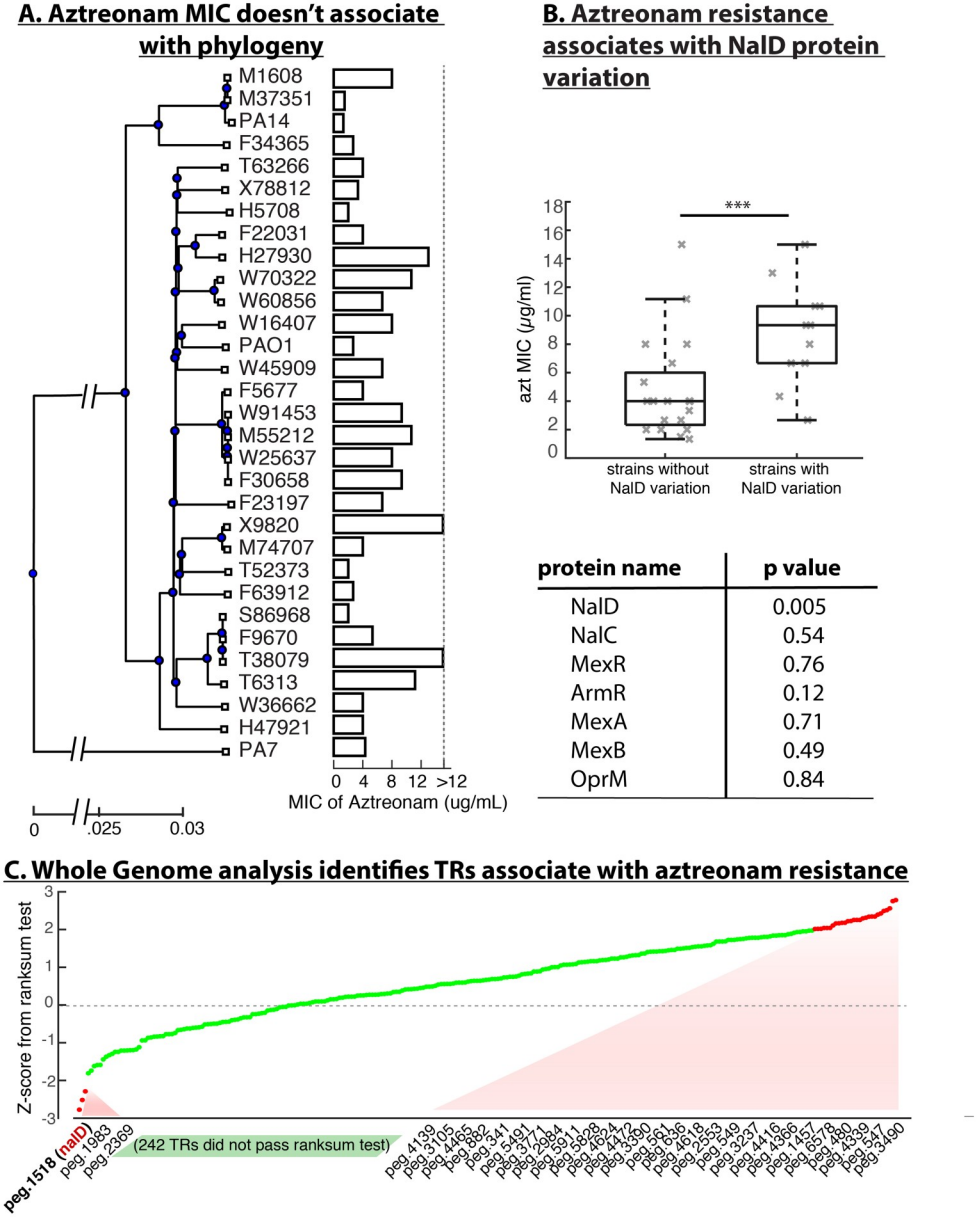
Aztreonam resistance is associated with variation in NalD across independent clinical isolates from acute *P*. *aeruginosa* infection. (A) Phylogenetic tree of isolates from acute infections of cancer patients reconstructed from core genes, including the type strains PA14, PAO1 and PA7. The minimal inhibitory concertation (MIC) of the aztreonam varies significantly across the phylogenetic tree, showing it is not a phylogenetically conserved trait. (B) NalD protein is the only protein in the *mexAB-oprM* efflux pathway that is strongly associated with aztreonam MIC in a rank sum test (***, p = 0.005). The table on the bottom shows the p-values for rank sum tests conducted on other proteins known from the *mexAB-oprM* efflux system and its regulatory pathway. (C) Expanding the analysis to all the transcriptional regulators encoded by the *P*. *aeruginosa* genome revealed 30 candidates whose protein sequences variation were associated with aztreonam MIC (see S2 Table), but NalD remained the strongest correlate.

To identify genomic features related to aztreonam resistance in patients acutely infected with *P*. *aeruginosa*, we started by measuring the minimum inhibitory concentration (MIC) of aztreonam in 31 *P*. *aeruginosa* isolates from cancer patients that we had previously sequenced [14]. Plotting the MIC levels next to a phylogenetic tree constructed from the core genome of the 31 isolates showed no discernable association between aztreonam MIC and phylogeny (Fig 1A). It is possible that the large genome-scale differences among the clinical isolates obscured the relationship between causal genetic variants and the desired phenotype. Therefore, we narrowed down the analysis to a smaller set of genes by focusing on the 19 genes where mutations emerged recurrently *in vitro* under aztreonam selection [13]. We tested the association between aztreonam MIC and the variation in the protein sequence coded by each of the 19 genes using a rank sum test (S1 Table). To our surprise, only one—NalD—passed the significance test (p = 0.0046), and associated with a >2-fold increase in average MIC.

NalD is a transcriptional repressor of the efflux system MexAB-OprM and mutations in NalD have been linked to multi-drug resistance, including to aztreonam [15,16]. However, the other two regulators of MexAB-OprM mutated in experimental evolution [13], NalC and MexR, were not significantly associated with aztreonam MIC in our clinical isolates (both with p>0.5). We then tested all the known proteins in the MexAB-OprM pathway [15,17] including the efflux pump coding proteins themselves (Fig 1B). Again, of the 7 proteins only NalD passed the association test. To confirm the association further, we downloaded NalD sequences of 126 *P*. *aeruginosa* isolates which had published aztreonam MIC values from the PATRIC database [18]. This collection, which has isolates from many sources including acute and chronic infections, showed again that NalD is significantly associated with aztreonam resistance (p<0.01).

This robust association suggested that mutations in NalD are main drivers of parallel evolution of aztreonam resistance in multiple lineages of *P*. *aeruginosa*. We compared the NalD protein sequences among our 31 clinical isolates including three type strains of *P*. *aeruginosa* (PA14, PAO1 and PA7). These NalD sequences are highly conserved, with only a few variations from the consensus (S1 Fig). Nonetheless, the strains that do vary from the consensus tend to rank high in terms of aztreonam MIC. One of the most resistant isolates, X9820, carries a copy of NalD with a deletion of residues 1~134 (>60% of the full length NalD) which plausibly causes loss of NalD function. Three other strains tested (W70322, W60856 and the type strain PA7) have mutations also in the 10^th^ alpha helix of the protein’s structure, and two strains (H27930, F23197) carry point mutation close to the C-terminus. Four strains (M55212, F30658, W91452, W25637) have mutation T11N located in the first residue of the first alpha helix, which likely impairs the DNA binding function of NalD.

Still, NalD variation alone explains only part of the aztreonam MIC. For example, two isolates that have the top aztreonam MIC, T38079 and T6313, have the same NalD sequences as the consensus (S1 Fig). Could variation in other transcriptional regulators explain aztreonam MIC? To address this question we comprehensively examined all annotated transcriptional regulators (>300) in the *P*. *aeruginosa* genome [18]. Thirty-one of these regulators were significantly associated with aztreonam resistance according to the rank sum test, but NalD still topped the list (Fig 1C).

### Evolution of *P*. *aeruginosa* aztreonam resistance within a patient

**Fig 2:**
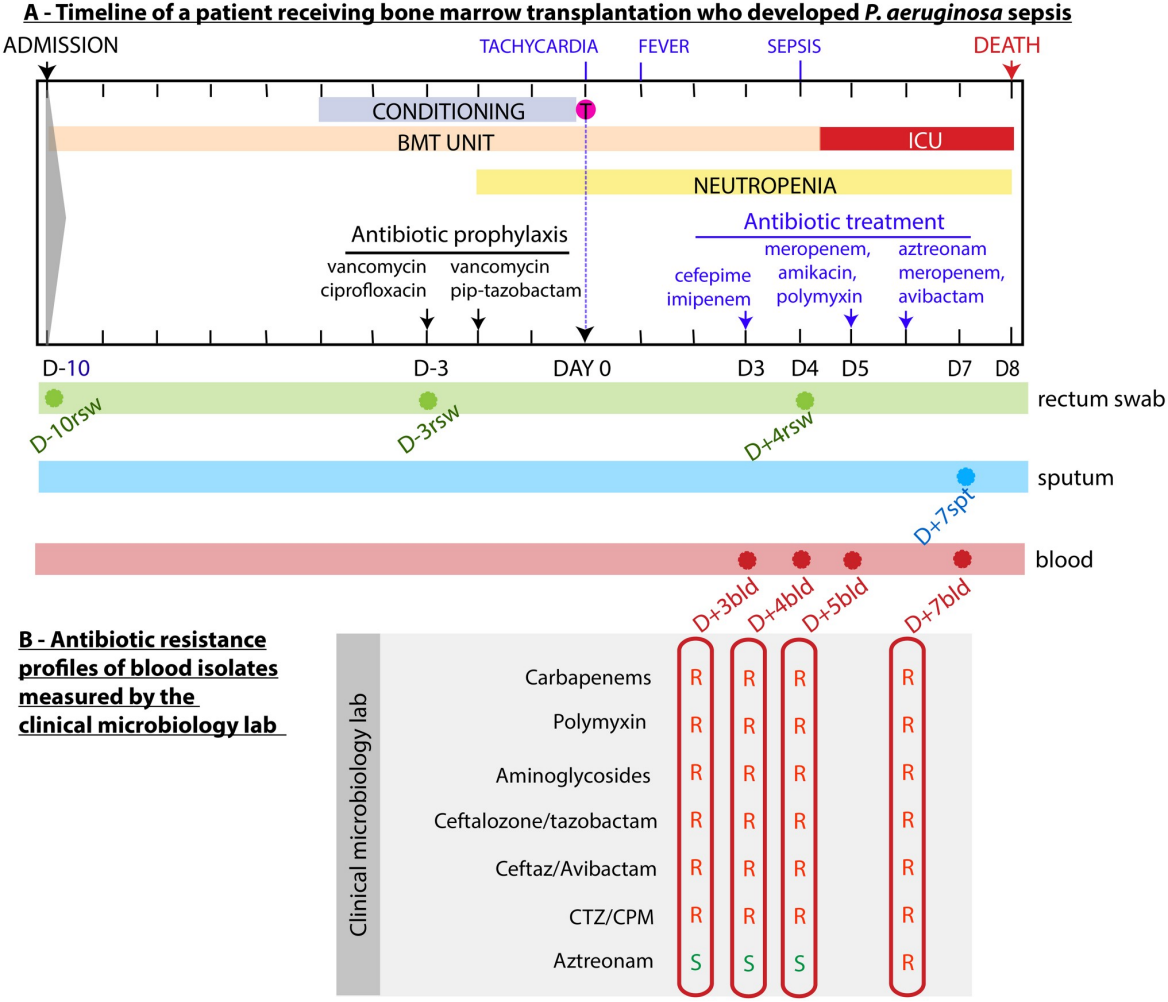
*P*. *aeruginosa* infection of a cancer patient hospitalized to receive hematopoietic cell transplantation evolved resistance to aztreonam in the course of therapy. (A) Timeline shows clinical events: conditioning regimen (myeloablation), hematopoietic cell infusion, location in the hospital (bone marrow transplantation unit [BMT] or intensive care unit [ICU]), the period of neutropenia, antibiotics administered, the day (relative to the day of transplant, day 0) and body site of origin (rsw: rectal swab; spt: sputum; bld: blood) of the eight *P*. *aeruginosa* isolates analyzed here. (B) Antibiotic resistance profiles of blood isolates measured by the clinical microbiology laboratory as the infection progressed; the profiles informed clinicians that the infection was multi-drug-resistant but also that it was initially sensitive to aztreonam (isolates D+3bld, D+4bld and D+5bld). As the disease progressed to become life-threatening sepsis, the patient was transferred to the ICU and was given aztreonam; however, the isolate D+7bld demonstrated resistance to aztreonam. The patient died on day +8.

To detail the drastic effects of aztreonam resistance *in vivo* we analyzed isolates obtained from a patient who died with an aztreonam resistant *P*. *aeruginosa*. The patient had been diagnosed with pre B cell acute lymphoblastic leukemia and was admitted (day -10 relative to day of transplantation) to Memorial Hospital for hematopoietic cell transplantation after undergoing first chemo remission (Fig 2A). As standard of care, the intense conditioning regimen compromises the patient’s immunity and can lead to life-threatening complications [19,20]. Therefore routine antibiotic prophylaxis with vancomycin, ciprofloxacin and pip-tazobactam was administered. This particular patient developed tachycardia on the day of stem cell infusion (day 0), followed by fever one day later (day +1). Blood cultures were drawn and cefepime and imipenem were administered to treat a plausible bacterial infection. On day +4 the patient worsened and developed sepsis, requiring transfer to the intensive care unit (ICU). On day +5, antimicrobials were changed to meropenem, amikacin and polymyxin. Blood cultures at this time tested positive for *P*. *aeruginosa* with resistance to multiple antipseudomonal agents but sensitive to aztreonam (Fig 2B). On day +6 the patient received aztreonam in addition to meropenem and avibactam as a last resort attempt to control the worsening infection. The patient’s clinical condition deteriorated and the patient eventually expired from sepsis on day +8.

To better understand the progression of aztreonam resistance, we tracked the origin of the *P*. *aeruginosa* infection by retrospectively culturing the initial rectal swab (day -10) and additional swabs taken at days -3 and +4, as well as a sputum sample from day +7. All samples produced *P*. *aeruginosa* colonies (Fig 2A). In total, we obtained eight *P*. *aeruginosa* isolates from this patient. We named those isolates by the number of days before (D-) or after (D+) the transplantation followed by body sites where they were isolated (Fig 2A). Importantly, the detection of *P*. *aeruginosa* on the day -10 rectal swab—obtained at the time of admission to the hospital—indicated that the patient had carried *P*. *aeruginosa* asymptomatically in the gut when entering the hospital. Of note, the patient’s pre-transplant care was delivered in another country and no prior rectal swab samples were available to determine the duration of carriage.

**Fig 3:**
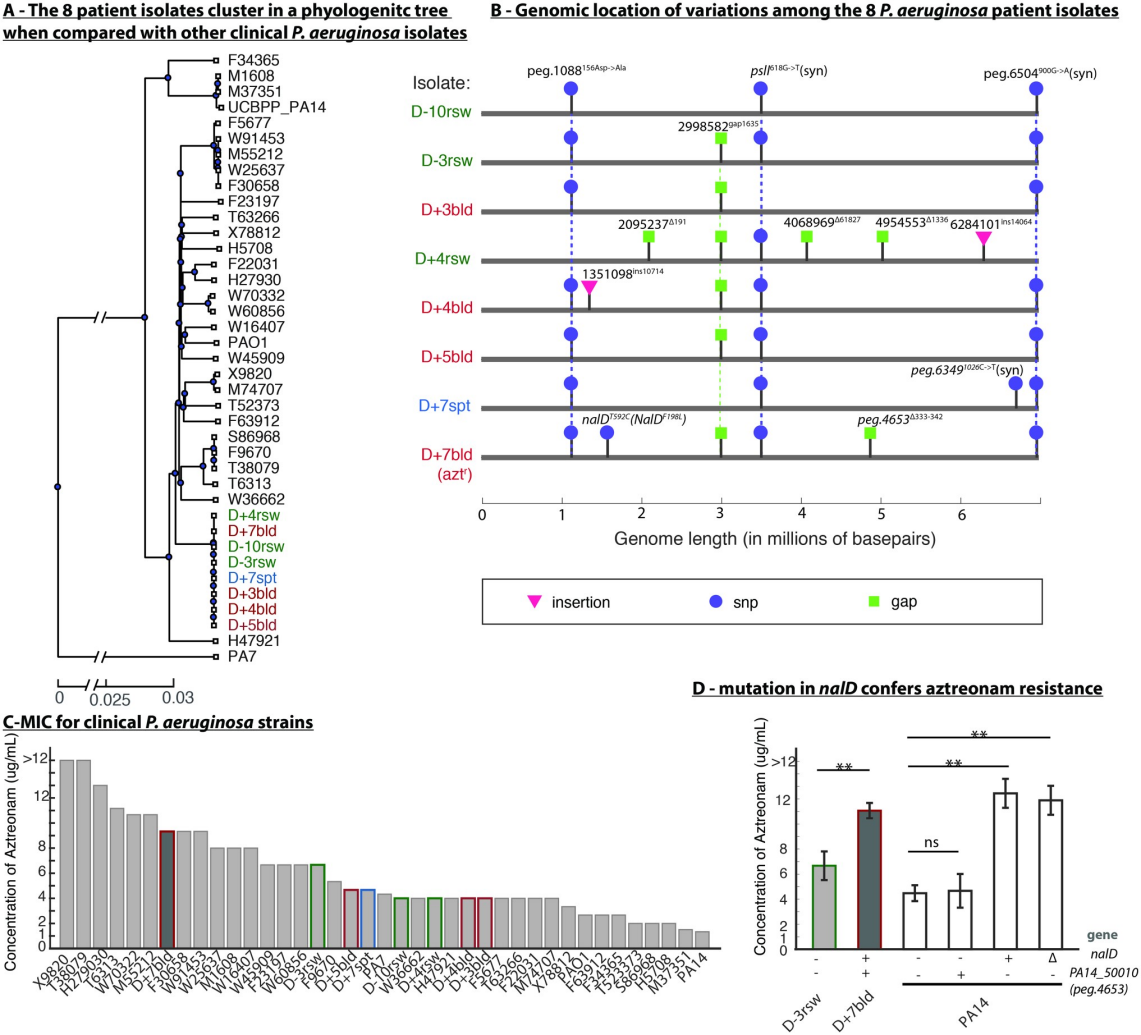
Whole-genome sequencing of eight *P*. *aeruginosa* isolates pinpointed the mutation NalD^F198L^ responsible for aztreonam resistance detected one day before the patient died. (A) A genome-based phylogenetic tree shows that the eight sepsis isolates are highly related to each other compared to other clinical isolates and type strains. (B) Genomic analysis revealed that the aztreonam-resistant isolate (D+7bld) had only two unique variations compared to aztreonam-sensitive isolates. Vertical dashed lines highlight the common presence of a given variation across multiple isolates. (C) Aztreonam MIC confirmed the clinical laboratory results for the eight sepsis isolates. (D) Experimental validation in PA14 showed that mutation in NalD but not in peg.4653 (PA14_50010) confers aztreonam resistance. “-”and “+” denote absence or presence of mutation found in D+7bld; “Δ” denotes deletion of the 10^th^ alpha helix in NalD protein. (**, p-value<0.01).

We sequenced the whole genomes of eight aforementioned *P*. *aeruginosa* isolates (hereafter called sepsis isolates as a group). To track whether the infection was originated from the patient or acquired from the hospital, we constructed the phylogenetic tree with the sepsis isolates and isolates from other cancer patients in the same hospital analyzed earlier [14], as well as the three type strains PA14, PAO1 and PA7 (Fig 3A). The tree revealed that the eight sepsis isolates belong to the PAO1 clade and are much more similar to each other than to any other isolates obtained earlier from the same hospital. This supports the notion that the infection progressed from a single clone that the patient harbored at the time of admission and was not acquired after admission to Memorial Hospital.

A genome alignment analysis revealed that the eight genomes are remarkably similar to each other (Fig 3B) with only a total of 12 unique allelic differences among them. We confirmed SNPs and small gaps using targeted (Sanger) sequencing (Materials and methods, S3 Table). Isolate D+4rsw is the most phylogenetically distinct strain among the sepsis isolates. Isolates D+5bld and D-3rsw are identical to each other and harbor only 4 variations from the ancestral alleles, which we inferred by using PAO1 as a reference. Among those variations are two discrete insertions greater than 10kb. A BLAST search in NCBI linked one to a transposon insertion (S6 Table) and another to a duplication of a region of its own genome encoding an unclear pathway. There is also a deletion homologous to a phage insertion (S6 Table).

Notably, the 12 genetic differences found among the eight isolates showed no pattern of association with either the time or the body site of isolation (Fig 3B), indicating that the *P*. *aeruginosa* population had diverged during colonization with multiple sub-clones coexisting at the same time in a single patient. Similar patterns of within host diversification were reported in other host-associated bacteria [21].

### Mutation in NalD conferred aztreonam resistance

To understand better why many clinical isolates have mutations in NalD, we first confirmed the mechanism of aztreonam resistance in the sepsis isolates. We conducted detailed measurement of aztreonam MIC for the eight sepsis isolates (Fig 3C, S1 Data). The aztreonam-resistant D+7bld isolate displayed a higher MIC (between 8–12μg/mL) than the other 7 sepsis isolates (<8μg/mL) consistent with the clinical report (Fig 2). However, D+7bld was not the isolate with the highest MIC when compared to the expanded collection comprising isolates from other cancer patients and the type strains PAO1, PA14 and PA7 (Fig 3C). Two isolates, X9820 and T38079, had MICs higher than 12μg/mL, the highest concentration tested. PA7, a type strain known for its resistance to a broad spectrum of antibiotics [22], showed similar aztreonam MIC to the 7 sepsis isolates that were considered clinically susceptible to that drug. The two widely used laboratory strains, PAO1 and PA14, had very low MICs.

The aztreonam-resistant sepsis isolate D+7bld has only two genetic variations from its most closely related isolates, D+5bld and D-3rsw. One of those two mutations is a 10bp deletion in a dehydrogenase of unclear function. To determine if this mutation alone could have increased *P*. *aeruginosa* resistance to aztreonam we introduced the same 10bp deletion in the corresponding dehydrogenase gene (PA14_50010, or peg.4653 in D+7bld) in the laboratory strain PA14 (Materials and methods, S4 Table). This mutation did not increase aztreonam MIC (Fig 3D, S2 Data).

The other mutation was a point mutation F198L found in NalD, a mutation that has not been reported nor selected through *in vitro* experiments before [13]. To confirm that this mutation alone could have caused aztreonam resistance we engineered the same NalD^F198L^ mutation into PA14 and the MIC increased 3-fold from 4μg/mL to 12μg/mL, a MIC similar to the MIC of the terminal sepsis isolate D+7bld (Fig 3D). This confirmed that the NalD^F198L^ mutation alone is sufficient for the observed aztreonam resistance in D+7bld, and is consistent with our finding that NalD mutation can increase aztreonam MIC on average by >2-fold (Fig 1B).

### NalD variation linked to multi-drug resistance

Our data suggests that NalD is a recurrent driver for aztreonam resistance in *P*. *aeruginosa* acute infection. NalD is not the only transcriptional regulator where mutations can drive antibiotic resistance. In cystic fibrosis patients treated with ciprofloxacin and azithromycin during chronic *P*. *aeruginosa* infection, mutations accumulate in transcriptional regulator NfxB, which negatively regulates another efflux pump, MexCD-OprJ [23]. Can mutations found in transcriptional regulators be used to predict the antibiotic resistance of a *P*. *aeruginosa* isolate? To address this question we posed two related but more specific questions: First, is there a way to predict aztreonam resistance from sequence variation in all transcription factors? Second, does NalD variation alone predict resistance to other antibiotics besides aztreonam?

**Fig 4:**
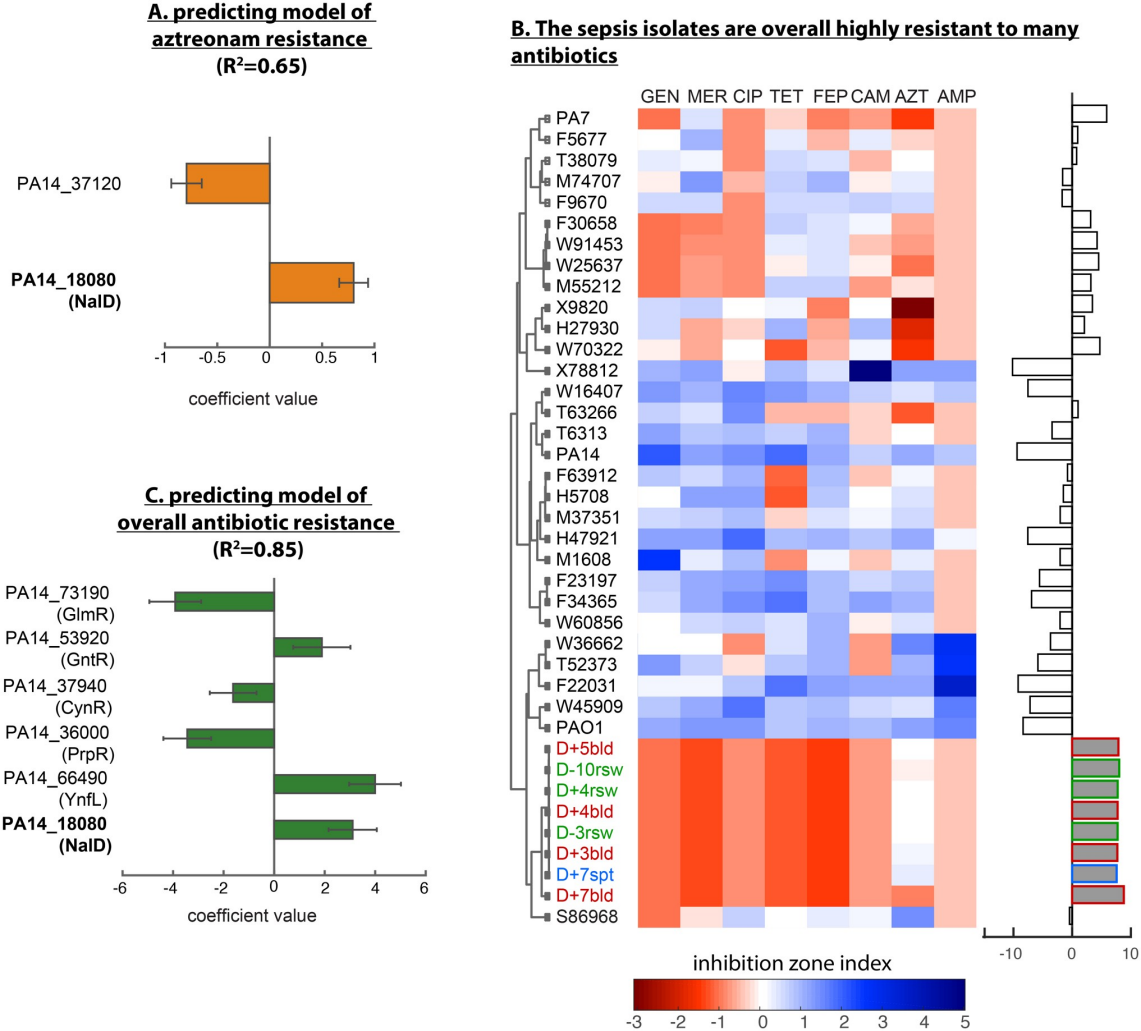
NalD mutation contributes to a general signature of antibiotic resistance. (A) A signature of aztreonam resistance obtained with LASSO regression shows only two transcriptional regulators, including NalD, and explains >60% of the variation in aztreonam MIC. The coefficients have units of fold-change. (B) Antibiotic inhibitions zones were measured using the disk assay for 8 antibiotics from several classes. The inhibition zone indices (shows as normalized areas of inhibition disk) show that the 8 sepsis isolates are resistant to multiple antibiotics. A resistance index computed from combining the negative values of the inhibition zone indices shows that the 8 sepsis isolates rank higher in multi-drug resistant than any other isolate tested. (C) The signature of multi-antibiotic resistance has six transcriptional regulators, including NalD, and explains >80% of the variation in the multi-drug resistance index. The coefficients have units of integrated fold-change across all the 8 antibiotics.

To answer the first question, we used a machine learning approach called LASSO (least absolute shrinkage and selection operator) [24]. We checked if this method could select transcriptional regulators based on their sequence variation to explain the aztreonam MIC data in our *P*. *aeruginosa* acute infection isolates. The LASSO produced a model where only two transcription factors (out of >200) explained more than 60% of variation in aztreonam MIC (R^2^ = 0.65, Fig 4A): NalD and PA14_37120, a probable LysR-type transcriptional factor. As expected, the coefficient for NalD was positive, implying that mutations in NalD tend to increase aztreonam MIC and therefore confer aztreonam resistance by >2x. By contrast, PA14_37120 had a negative coefficient, indicating that isolates that contain PA14_37120 different from the consensus have lower aztreonam MIC and, therefore, tend to be more sensitive to aztreonam. The negative relationship seems to be common for LysR-type proteins which are positive regulators of enzymes that degrade antibiotics [25], suggesting that this specific type of transcriptional factors could potentially be explored as a target to sensitize *P*. *aeruginosa* to aztreonam.

To answer the second question, we measured the sensitivities of each *P*. *aeruginosa* acute infection isolate to a panel of eight antibiotics from several classes (ciprofloxacin, gentamicin, aztreonam, chloramphenicol, ampicillin, tetracycline, meropenem, cefepime) and quantified the degree of multi-drug resistance by combining those sensitivity values into a multi-drug resistance index (Materials and methods, Fig 4B, S3 Data). Notably, the group of eight sepsis isolates showed the highest multi-drug resistance index among all strains. A LASSO analysis identified six transcriptional regulators that combinatorically explain more than 80% of the variation (R^2^ = 0.85) in the multi-drug resistance index (Fig 4C, S7 Table). Strikingly, NalD arose again as a strong contributor to this signature. To evaluate the contribution of NalD to the resistance to drugs other than aztreonam, we removed aztreonam from the multi-drug resistance index and re-ran LASSO regression (S4 Fig). NalD remained an important contributor to this multi-drug resistance signature, suggesting that mutation in this transcription factor is relevant for general resistance, not just to aztreonam. These results agree with the broad substrate specificity of the MexAB-OprM efflux system [26] and with a previous finding that aztreonam selection can result in collateral resistance to antibiotics including tobramycin, colistin and ciprofloxacin [13]. In addition, a transposon mutant in another regulator identified by our LASSO analysis but with negative coefficient, GlmR, was hypersusceptible to a range of antibiotics in *P*. *aeruginosa* strain PAO1 [27], suggesting its important role in the development of multi-drug resistance.

### NalD structure indicates mechanism of efflux upregulation

**Fig 5:**
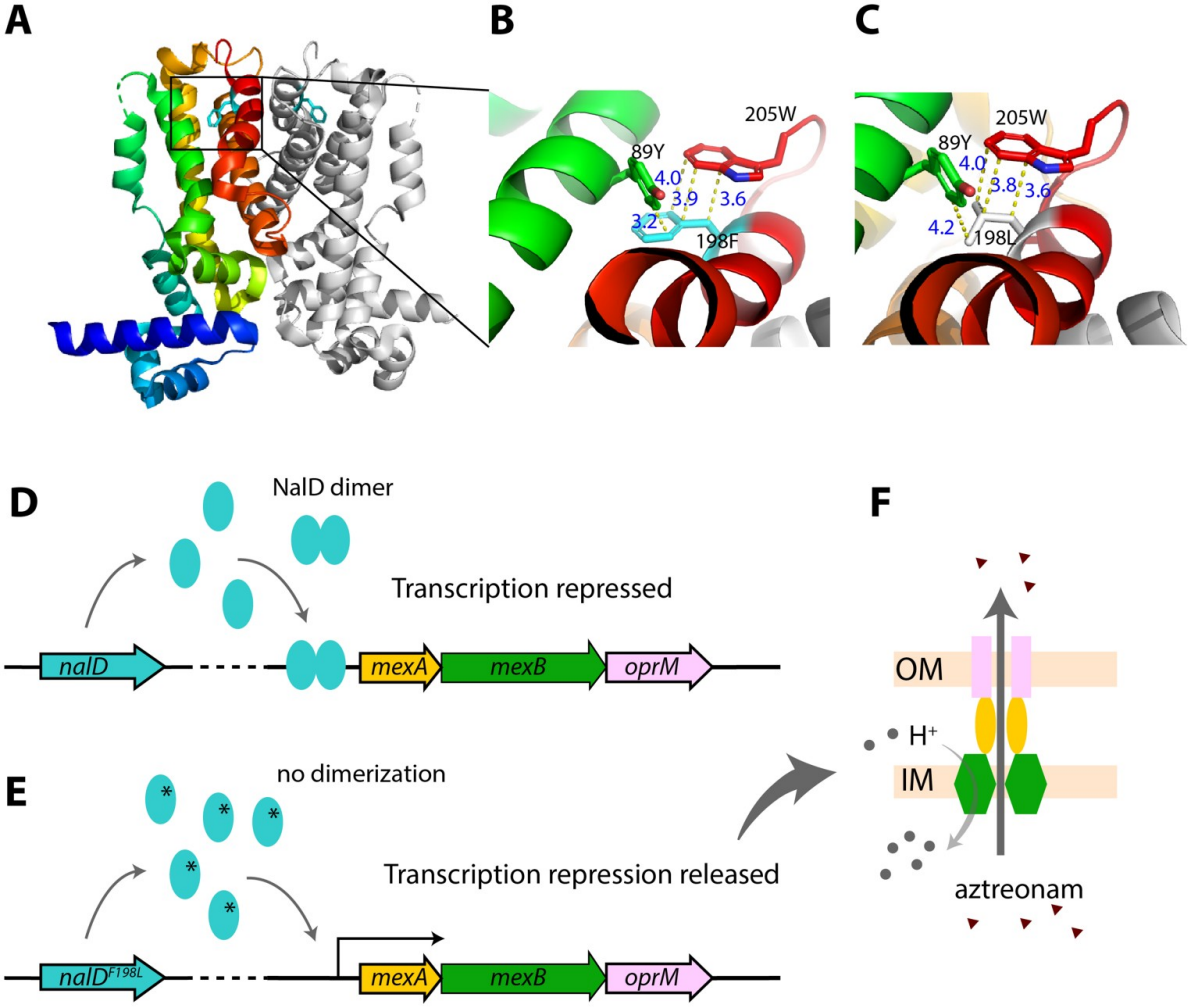
Molecular details of mechanism of acquired aztreonam resistance in the sepsis patient infected with *P*. *aeruginosa*. (A) 3D structure of NalD dimer (PDB id: 5daj) with residue 198F (phenylalanine) shown as stick model. One copy of NalD is rainbow colored, while the other is in gray. The 10^th^ helix is shown in red. (B) A closer look of residue 198F and possible interaction with two nearby aromatic residues, 89Y (tyrosin) and 205W (tryptophan). Those three residues are close together and could have aromatic interactions or a possible hydrogen bond (3.2 Å) between 198F and 89Y. 198F also aligns well with 205W, both of which have ring structure and could form a displaced pi stacking that stabilizes the NalD structure (3). (C) Prediction of mutation effect based on NalD structure. The mutation F198L would widen the distance between carbon groups and lose the *pi-pi* interaction, which could ultimately destabilize NalD dimerization. (D) Wild-type NalD dimer represses transcription of *mexAB*-*oprM* operon. (E) The mutation NalD^F198L^ could interfere with dimerization, and de-repress transcription. (F) MexAB and OprM form an anti-porter system that exports aztreonam, increasing resistance [29].

To investigate how the NalD^F198L^ mutation alters NalD protein function, we studied a high-resolution crystal structure of NalD protein from *P*. *aeruginosa* PAO1 (PDB id: 5daj) [28], which has the same sequence as the NalD of D+7bld except for the mutation identified in this study. Structural analysis showed that the residue F198 lies in the 10^th^ alpha helix, which locates in the interface of the NalD dimer (Fig 5A). This residue is close to two other residues in tertiary structure: 205W and 89Y (Fig 5B). All of these three residues have aromatic rings and the interaction between them could be strong, such as pi-stacking, and stabilize the 10^th^ alpha helix facing the dimerization interface of NalD. Changing the residue 198 from F into L (Fig 5C) likely impairs these aromatic interactions and destabilizes the 10^th^ alpha helix, impacting dimerization and further de-repressing *mexAB-oprM* (Fig 5D–5F).

To validate this model, we deleted the 10th alpha helix of NalD in PA14 without shifting its reading frame (Materials and methods, S4 Table). The deletion increased aztreonam resistance of PA14 to the same level of the PA14 carrying NalD^F198L^ and the sepsis isolate D+7bld (Fig 3D). Therefore, the mutation NalD^F198L^ could indeed have conferred aztreonam resistance by loss of function and release of *mexAB-oprM* expression.

### Acquisition of aztreonam resistance shows no fitness cost

The D+7bld isolates acquired aztreonam resistance through a point mutation in NalD, which possibly derepressed the expression of an efflux system. Would this mutation carry a fitness cost in the absence of the antibiotic? To answer this question, we first cultured D+7bld and D+5bld individually *in vitro* without aztreonam, where they showed the same growth rate (S2 Fig). We then asked if the D+7bld would be outcompeted by D+5bld when cultured together. In a competition experiment, we initially mixed D+7bld:D+5bld (1:1000) in a liquid media without aztreonam (S1 Text); we observed no change in that initial frequency, which confirmed that there is no fitness cost in the absence of the antibiotic (S3 Fig). By contrast, in the presence of aztreonam the NalD mutation confers a huge competitive advantage: when 2μg/mL or 4μg/mL aztreonam was added to the mixed population, the frequency of D+7bld increased ~10 fold and >200 fold respectively (S3 Fig). These results suggest that the NalD mutation in the absence of aztreonam either did not have direct fitness cost or the cost has been compensated for by other mechanisms. Possible mechanisms included the secondary 10bp deletion in a dehydrogenase, a non-mutational mechanism that changed bacterial physiology globally or through changes in specific pathways.

**Fig 6:**
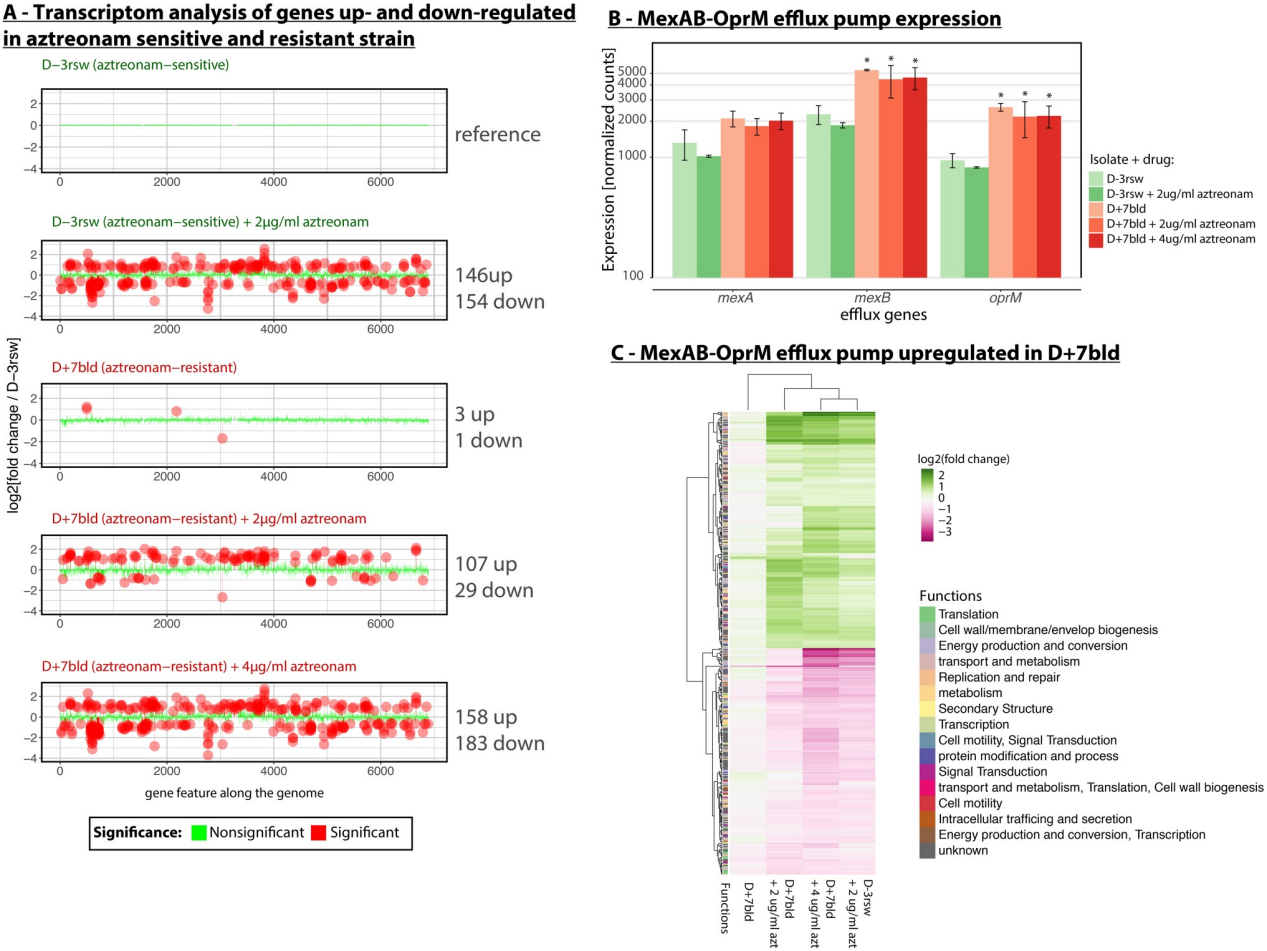
RNA-seq analysis shows that the aztreonam-resistant isolate D+7bld up-regulates the *mexAB-oprM* efflux system and attenuates response to aztreonam stress. (A) We compared the transcriptomes to reference isolate D-3rsw at 0μg/mL of aztreonam and found hundreds of differentially expressed genes. (B) The up-regulation of the *mexAB-oprM* efflux system in D+7bld supported that the NalD mutation released the transcriptional repression of *mexAB-oprM*. (C) The transcriptome of D+7bld at 4μg/mL aztreonam resembled the transcriptome of the aztreonam sensitive D-3rsw at half that dose (2μg/mL), confirming that the aztreonam resistance allows the strain to sustain higher levels of antibiotic challenge. azt, aztreonam. *, p-value<0.05.

To understand how the NalD mutation conferred resistance without a fitness cost, we compared the transcriptome of the NalD-mutated D+7bld to the susceptible D-3rsw, which differed from D+7bld by only two mutations. During exponential growth without antibiotics, only four genes were significantly differentially-expressed between those two isolates after multiple hypothesis correction (absolute log2-fold change ≥0.5 and adjusted p-value≤0.05). Two of those genes were *mexB* and *oprM* (Fig 6A), the genes coding for the inner and outer membrane components of the efflux pump. We then analyzed the transcriptomes of both isolates in the presence of different concentrations of aztreonam. Hundreds of genes showed significantly differential expression, as expected from the stress of antibiotic exposure [30]. D+7bld had less differentially-expressed genes than D-3rsw (136 compared to 300) at the sub-lethal aztreonam concentration of 2μg/mL. When the concentration of aztreonam increased to 4μg/mL—a level lethal to D-3rsw but not to D+7bld—the differentially-expressed genes in D+7bld increased to 341, a level of response that is similar to D-3rsw at 2μg/mL aztreonam (Fig 6A). A closer examination of the *mexAB-oprM* operon showed that none of the operon genes changed their expressions in D-3rsw exposed to aztreonam (Fig 6B). The efflux system was, however, over-expressed in D+7bld for all antibiotic concentrations (Fig 6B). Our transcriptomic data support the canonical model whereby the mutation in NalD released the repression of the *mexAB-oprM* operon regardless whether aztreonam was added to the medium or not [15] (Fig 5E).

We clustered expression levels of genes that are differentially expressed in at least one condition relative to the reference (D-3rsw, no aztreonam). The transcriptome of D+7bld was not much different from the D-3rsw in the absence of aztreonam. The overall expression profiles compared between D+7bld at 4μg/mL and D-3rsw at 2μg/mL of aztreonam were indeed similar to each other (Fig 6C), suggesting that the overexpression of *mexAB-oprM* had a dampening effect on the response to the antibiotic. The profile of D+7bld at 2μg/mL lies in between the profile of no aztreonam and of 4μg/mL aztreonam, further supporting that aztreonam induces a dose-dependent cellular response.

### Integration of metabolic network model with transcriptomics data accurately predicts bacterial growth

Among the differently transcribed genes identified above, the top 3 groups of 107 functionally annotated genes are “transport and metabolism” (73 genes), “energy production and conversion” (54 genes), and “metabolism” (43 genes) (Fig 6C, S4 Data), suggesting a link between bacterial metabolism and aztreonam resistance. Metabolic fluxes can be impacted by the transcription of metabolic genes [31]. Therefore we sought to infer flux changes on the basis of gene expression changes using a computational model of the metabolic network. To study our sepsis isolates, we used a high-quality genome-scale model of *Pseudomonas* metabolism, iJN1411 [32], which contains 1411 gene products and 2826 reactions. By combining the RNA-seq data with the model, we aimed to explore how differential gene expression redistributed the metabolic fluxes and pathway usages.

The method for integrating transcriptomic data with the iJN1411 model involves two major steps (Materials and methods): (1) building a reference model for D-3rsw at 0μg/mL aztreonam using transcriptomics data in the reference condition, and (2) modifying the reference model to accommodate gene expression changes measured for D-3rsw at 2μg/mL and D+7bld at 0, 2, 4μg/mL aztreonam. Under the reference condition, we approximated the flux bounds of reactions in the iJN1411 model by the optimal flux distribution that is most consistent with the mRNA levels in that condition, thereby constraining the metabolic solution space (i.e., range of feasible steady-state fluxes) to represent the actual metabolic behavior implied by data. To build metabolic models in other conditions, we incorporated the transcriptional differences between these conditions and the reference condition by multiplying the reconstructed flux bounds of each reaction in the reference model by expression fold-change values of corresponding genes associated with each reaction.

**Fig 7:**
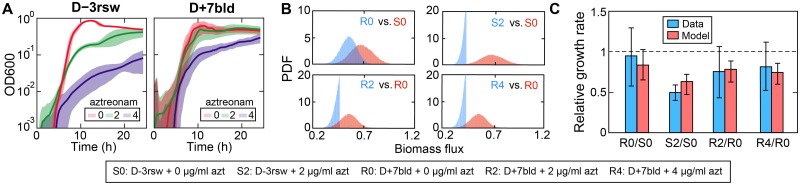
Validation of metabolic model using experimental growth data. (A) Experimental growth curves of both sensitive (D-3rsw) and resistant (D+7bld) *P*. *aeruginosa* strains at various aztreonam concentrations (μg/mL). (B) Steady state distribution of biomass flux predicted from metabolic models for the same experimental conditions (except for D-3rsw at 4μg/mL aztreonam). PDF: probability density function. (C) Comparison of the measured growth rate (blue bars) with the model predictions (red bars). The measured growth rates were obtained by fitting an exponential growth model to the exponential phase of the growth curves shown in (A). The predicted growth rates were approximated from the mean of the biomass flux distributions shown in (B). The growth rates are relative to that of the sensitive strain in the absence of aztreonam (S0). Error bars: standard deviation.

The resulting 5 metabolic models were validated by comparing the growth rates measured experimentally at various aztreonam concentrations (Fig 7A) to model predictions (Fig 7B). Simulations using flux sampling showed that the distributions of biomass flux (i.e., flux through biomass production reaction) between D-3rsw and D+7bld in the absence of aztreonam overlapped, suggesting that the growth capacity of the resistant strain is likely uncompromised by the development of aztreonam resistance. This is consistent with our finding above that the sensitive strain did not outcompete the resistant strain *in vitro*. However, their biomass flux distributions with aztreonam present were truncated and heavily skewed to the left, indicating that the transcriptomic responses to aztreonam heavily restrict their growth rates. Using biomass as a proxy of bacterial growth, we showed that the ratios of predicted mean biomass flux (Fig 7C, **red bars**) agree qualitatively with the experimentally measured growth rates (Fig 7C, **blue bars**).

### Modeling-based analysis reveals metabolic adaptations in the resistant strain

**Fig 8:**
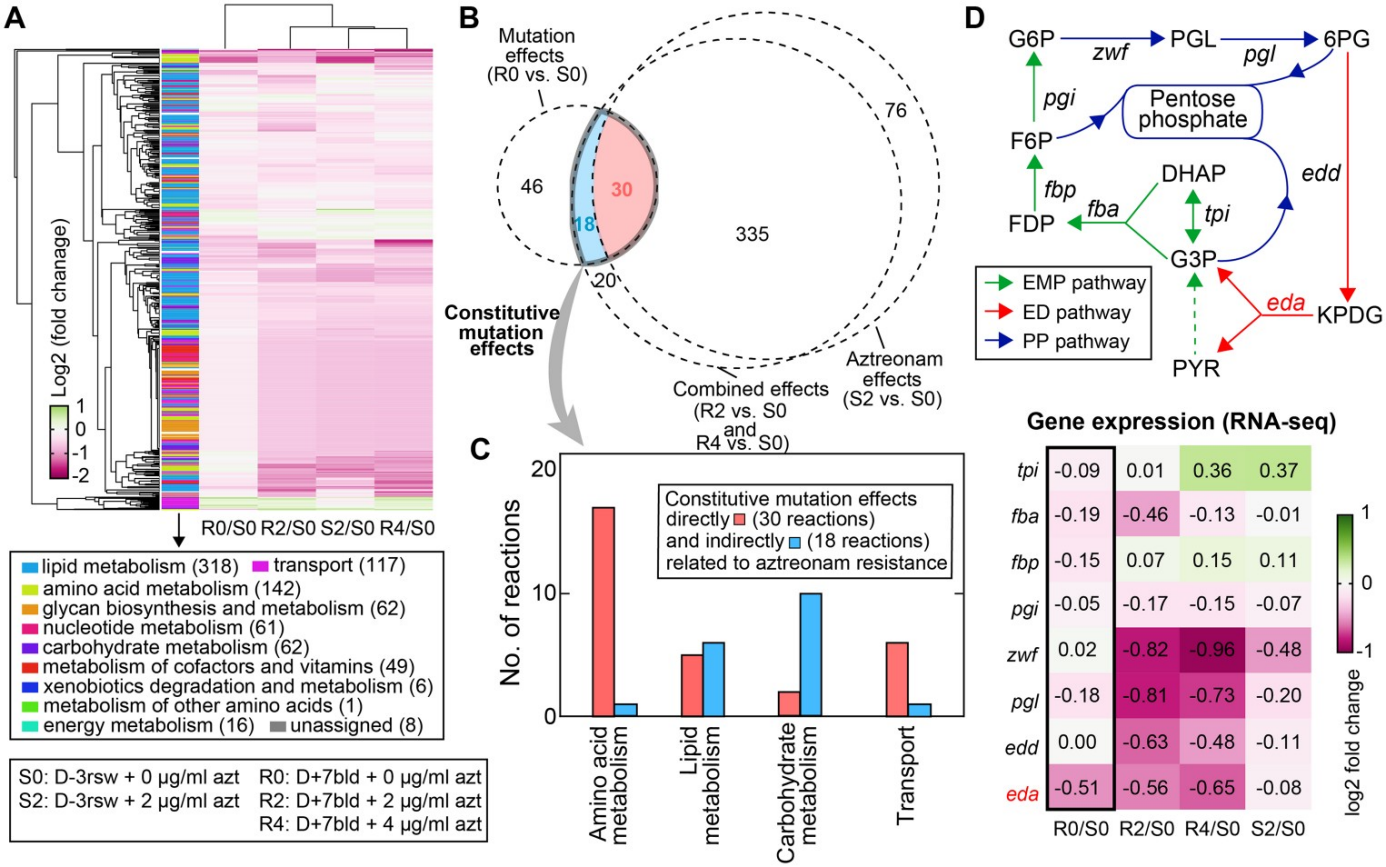
Evolved metabolic-level adaptations in the aztreonam-resistant strain. (A) Metabolic flux changes relative to the reference condition S0 (D-3rsw, no aztreonam). (B) Venn diagram showing the overlap between reactions whose flux levels significantly altered by aztreonam alone, mutations alone, and their combination. Among all 48 reactions constitutively modulated by mutations (i.e., constitutive mutation effects), 30 are directly related to aztreonam resistance (because aztreonam can induce their responses) and 18 are indirectly related. (C) Grouping of the 48 constitutively modulated reactions by pathways they belong to. (D) Expression of *eda* serves as a bottleneck to the flux through the Entner-Doudoroff (ED) pathway (red arrows). The connected Embden-Meyerhof-Parnas (EMP) pathway (green arrows) and pentose phosphate (PP) pathway (blue arrows), as well as the relative expression changes of the major genes in the three pathways (heatmap) are also shown. *Tpi*: triose phosphate isomerase; *fba*: fructose-1,6-biphosphate aldolase; *fbp*: fructose-1,6-biphosphatase; *pgi*: glucose-6-phosphaate isomerase; *zwf*: glucose-6-phosphate dehydrogenase; *pgl*: 6-phosphogluconolactonase; *edd*: phosphogluconate dehydratase; *eda*: 2-dehydro-3-deoxy-phosphogluconate aldolase; DHAP: dihydroxyacetone phosphate: FDP: D-fructose-1,6-biphosphate; F6P: fructose-6-phosphate; G6P: glucose-6-phosphate; PGL: 6-phospho-D-glucono-1,5-lactone; 6PG: 6-phospho-D-gluconate; KPDG: 2-dehydro-3-deoxy-6-phospho-D-gluconate; G3P: glyceraldehyde 3-phosphate; PYR: pyruvate.

Using the validated models, we first assessed the metabolic flux changes across the conditions of different strains and aztreonam concentrations (Fig 8A). For each condition, we calculated the flux through each reaction as the median of its distribution obtained by uniformly sampling the corresponding solution space 100,000 times. This is different from a typical flux balance analysis which optimizes a presumed objective function. We chose this method because biological organisms operate under multiple competing objectives related to fitness (e.g., maximal growth, fast adaptive response) [33]. Antibiotic challenge may introduce new objectives, making any single objective function inappropriate to describe the metabolic goal of bacterial cells.

Metabolic flux changes shown above can be induced by either acquiring mutations, adding aztreonam to the media, or combination of both. In the absence of aztreonam, the resistant strain only displayed 12% change of metabolic fluxes relative to the sensitive strain (absolute flux value >10^−3^, absolute log2-fold change ≥0.5 and adjusted p-value <0.05, Materials and methods). Adding aztreonam induced a system-wide flux rearrangement for both strains: over 50% of all 844 reactions with active fluxes were significantly up- or down-regulated. Our result thus suggests a much weaker metabolic effects caused by mutations compared to aztreonam, which agrees with transcriptomic data and could explain the lack of fitness cost of D+7bld observed in experiments. A Venn diagram (Fig 8B) illustrates the overlaps of reactions whose flux levels were significantly changed by mutations alone (flux changes between the sensitive and resistant strain in the absence of aztreonam), by aztreonam alone (flux changes in the sensitive strain between w/ and w/o aztreonam), as well as by their combination (flux changes between the resistant strain with aztreonam and the sensitive strain without aztreonam). We found 403 reactions affected by both factors (i.e., the combination effects), among which 335 can be perturbed by aztreonam as the sole factor, indicating again that aztreonam causes the majority of flux changes when both factors are present.

The Venn diagram also reveals how the resistant strain rewired metabolic fluxes as secondary effects of the NalD mutation beyond its primary function that releases MexAB-OprM efflux pump. There are 48 reactions in total (30 constitutive mutation effects, and 18 aztreonam resistance effects) displaying significant flux changes between the resistant and sensitive strain regardless of the presence and concentration of aztreonam (Fig 8B, S5 Data), which indicates that those 48 reactions are not directly related to aztreonam triggered growth defects as the rest reactions do. These constitutive metabolic adaptations include the secondary mutation effects that may or may not be related to aztreonam resistance. 30 reactions that are also affected by aztreonam in the sensitive strain likely provide the mechanisms that enable *P*. *aeruginosa* to resist the action of aztreonam. They were all downregulations and found in amino acids, lipid, carbohydrate metabolism as well as membrane transport system (Fig 8C). This finding is consistent with a previous study showing that aztreonam perturbed the metabolite levels in the same pathways as in another Gram-negative, nosocomial pathogen *Acinetobacter baumannii* [34]. The major identified reactions involved in amino acid metabolism are related to branched-chain (BCAA: leucine, isoleucine and valine) and aromatic amino acids (AAA: phenylalanine, tyrosine). Additionally, 4 out of 6 transport reactions are associated with uptake of valine and phenylalanine, further linking transport and utilization of BCAA and AAA to aztreonam resistance.

The other 18 reactions that are not affected by aztreonam in sensitive strain may suggest mechanisms that do not contribute to the mechanism of resistance but compensate for its associated fitness costs. They were all downregulated reactions as well, among which we found two reactions (mediated by EDA and EDD) from the Entner-Doudoroff (ED) pathway (Fig 8D, **red arrows**) in carbohydrate metabolism. By examining the transcriptional level of enzymes in the central carbon metabolism, we determined that it is *eda*, a gene encoding KPDG (2-dehydro-3-deoxy-phosphogluconate) aldolase, but not any other enzyme-coding genes, that acts as the bottleneck to the pathway flux in the resistance strain because its expression was constitutively downregulated by mutations regardless of aztreonam (Fig 8D).

## Discussion

*P*. *aeruginosa* is a major pathogen with a large genome, and extensive genomic variation among the strains in the same species. The high genomic diversity challenges our ability to predict clinically important phenotypes, particularly antibiotic resistance. Whole genome sequencing of *P*. *aeruginosa* isolates from cystic fibrosis patients had already revealed adaptations to the pressures experienced in the chronically-infected lung [6] and antibiotic therapy [23] but the adaptations to the pressures experienced in acute infection remained less clear. Acute infections may start when *P*. *aeruginosa* translocate from the environment, from another patient or, as we have seen here, from asymptomatic colonization in the patient’s own microbiome. Systems-level analyses can help us understand how these transitions shape *P*. *aeruginosa* physiology and impact its broad response to antibiotics. Here we used mathematical models to assist in the interpretation of antibiotic resistance from sequenced genomes. Application of these methods to predict antibiotic resistance in a clinical setting will likely require a better understanding of genetic function and gene interaction networks beyond our present knowledge. Evolution experiments conducted in the laboratory can help uncover some of these mechanisms, but such conditions are perhaps drastically different to those experienced in human infection [35]. Here we show that specific clinical cases can help bridge the gap between laboratory insights and clinical relevance.

We identified that recurrent mutation in NalD is associated with resistance to aztreonam and to other antibiotics in patients acutely infected with *P*. *aeruginosa*. We dissected the case of a multi-drug resistant strain that escaped from the patient’s gut microbiota into their bloodstream, acquired the NalD mutation and ultimately killed the patient. The patient came to Memorial Sloan Kettering (located in the USA) from another country, where use of antibiotics without prescription is more common. Prior use of antibiotics likely explains the unusually high antibiotic resistance of all isolates obtained from this patient (Fig 4B). The translocation of the *P*. *aeruginosa* from an asymptomatic gut colonizer to the bloodstream agrees with previous studies showing that disruption of the commensal gut microbiome with antibiotics increases the chance of bloodstream infections by antibiotic resistant bacteria residing in the gut [36,37].

The molecular events we uncovered agree with the known mechanism where a NalD loss-of-function mutation releases *mexAB-oprM* expression and confers resistance. The wild type NalD responds to inducers such as novobiocin [28]. The strain D-3rsw, which carries the most common (wild type) NalD sequence, did not increase *mexAB-oprM* expression in 2μg/mL of aztreonam (Fig 6B), which suggests that NalD does not respond to aztreonam. It makes sense in light of *P*. *aeruginosa* evolutionary history that the wild type NalD is unadapted to respond to this antibiotic, which is a relatively recent synthetic drug [11–13] that was probably absent in the evolutionary history of *P*. *aeruginosa*. Previous experiments had shown that loss of function in transcriptional regulators offers a quick way for bacteria to adapt to such new challenges [38]. Our data shows that the NalD point mutation can occur in a patient and cause a rapid increase in drug resistance, even while a patient receives treatment.

The rapid adaptation of nosocomial pathogens often results from mutations in transcriptional regulators [6,39–44]. This is perhaps expected: mutations in transcriptional regulators provide the most dramatic and rapid means to change bacterial physiology [38]. Their relationship to antibiotic resistance may be less well understood [45], especially in acute infections where disease progression and transmission can happen quickly, and the secondary effects of mutations are often obscured by the primary effects and making them barely detectable. However, secondary effects can be critical: they can reduce the fitness cost of resistance mutations and can even help provide collateral resistance to other antibiotics [29].

In this study, we investigated the secondary effects of the NalD mutation by integrating metabolic modeling and transcriptomics data. Over the last decade, metabolic network analysis that combines genome-scale metabolic models and omics data have been applied to study antibiotic resistance in bacteria and to suggest therapeutic targets [46–50]. Although the molecular (primary) function of the NalD mutation has been widely studied, our work adds to our limited understanding of its secondary effects. Our method indicated 48 reactions that may be constitutively downregulated in the aztreonam resistant strain. This is consistent with a general notion that drug resistance is associated with reduced, rather than enhanced, cell metabolism. We predicted that metabolic changes in the membrane transport and metabolism of BCAA and AAA are directly connected to the development of aztreonam resistance. Previous studies have suggested that the carbon catabolite control system CbrAB/Crc regulates BCAA uptake and utilization [51] as well as antibiotic resistance in *P*. *aeruginosa* [52–54]. Since channels that actively uptake amino acids can also transport antibiotics with sufficient structural similarity (e.g., *Escherichia coli* glycine transport system can also uptake the antibiotic D-cycloserine [55]), aztreonam resistance can be possibly potentiated by decreasing drug uptake through BCAA transporters via the CbrAB/Crc system, in addition to the efflux provided by the upregulated MexAB-OprM. Using a defined synthetic cystic fibrosis sputum medium, AAA were reported to induce biosynthesis of the *Pseudomonas* quinolone signal (PQS) [56], a quorum-sensing signaling molecule that regulates up to 12% of the *P*. *aeruginosa* genome [57]. A PQS mutant is more tolerant to ciprofloxacin than its wild-type [58], which supports that downregulating the AAA pathway may protect *P*. *aeruginosa* from aztreonam by reducing its PQS level.

We also predicted metabolic changes in ED pathway as a potential compensatory mechanism that reduces costs associated with the NalD mutation. Normally, the ED pathway is alternative to glycolysis and catabolizes glucose to pyruvate. However, when growing on casamino acids, *P*. *aeruginosa* must operate through gluconeogenesis to produce several essential metabolite precursors such as fructose-6-phosphate (FBP) and glucose-6-phosphate (G6P) for biomass production. The gluconeogenic flux is funneled into the oxidative branch of the pentose phosphate pathway and the ED pathway, forming a cyclic loop (known as the EDEMP cycle [59] that starts and ends with pyruvate) (Fig 8D). The recycling of hexoses back to trioses through the ED pathway can provide two potential compensatory mechanisms. First, it provides a reservoir flux and its downregulations can redirect the flux towards desired pathways. For example, reduced flux through the ED pathway can compensate for decreased flux from F6P to pentose phosphate pathway and biosynthesis of peptidoglycan, where the latter is the direct target for aztreonam. However, this compensatory effect may be limited if the reservoir flux is small. From another angle, a small ED pathway flux can rapidly become depleted in adverse environmental conditions and thus possibly acts as a sensor to indicate the hardship of the environment that *P*. *aeruginosa* faces. The functioning of the environment sensor will require the cooperation from a flux-signaling metabolite, which translates the flux change to change in metabolite level and stimulates specific pathways to combat the hardship [60]. The potential distant regulatory role of ED pathway has been implicated in another human pathogen, *Vibrio cholerae*, where activation of the ED pathway leads to higher transcriptional levels of the prime virulence genes [61].

Our computational investigation of *P*. *aeruginosa* metabolism generates new hypothesis for future research but has noteworthy limitations. First, we used the metabolic model iJN1411 which was developed for *P*. *putida*, a species very close to *P*. *aeruginosa*. We chose this model because of its outstanding quality: the model has 2826 reactions constructed from 409 citations and 72% of the reactions are supported by at least one reference [26]. It is important to keep in mind that the non-conserved pathways between those two species may lead to different metabolic flux distribution. Nonetheless, we expect the effect of metabolism difference to be minor because about 80% of the 1411 genes in model iJN1411 were covered by our transcriptomics. Second, no matter how many times we sample the metabolic space in the reference model, it is always possible that the optimal solution of highest consistency with gene expression may not be unique and alternative solutions that are equally optimal can exist. Third, all methods that use transcriptomics for metabolic modeling have a major limitation: metabolic flux and transcriptomics are only loosely correlated. Metabolic fluxes depend not only on the mRNA levels of the enzyme that catalyzes each reaction, but also on many factors including post-transcriptional modulations and allosteric regulations [62,63]. In the future, these limitations could be overcomed by a high-quality *P*. *aeruginosa*-specific metabolic model, assisted by metabolic flux data to constrain solution space.

## Materials and methods

### Ethics statement

According to the NIH guide for Human Subjects Research, this work is “exempt from the human subject’s regulations, category 4 (Exemption 4)” because it involves “only the use of secondary analysis of biological material/tissue/specimens or data not collected specifically for this study” and “the specimens or data previously collected are de-identified for the purpose of this study by someone involved in the research study. For example, your collaborator will provide you with aliquots of specimen that are no longer linked to the subject identifiers or you are extracting clinical data from medical records without retaining the subject name or medical record number.”

### Microbiological methods

Bacterial culture, gene mutagenesis and genomic sequencing were performed as previously described [14] and more details are given in S1 Text. Primers are listed in S3 and S4 Tables. Other detailed experimental methods including antibiotic resistance assay, bioinformatics and transcriptomic assay are included in **SI** as well.

### Aztreonam susceptibility test

In clinical lab, phenotypic antimicrobial susceptibility testing (AST) was performed by broth microdilution using the Gram-Negative MIC Panel type 43 on the MicroScan WalkAway system (Beckman Coulter) following overnight incubation and photometric determination of bacterial growth. AST results of aztreonam for *P*. *aeruginosa* were interpreted using the Clinical and Laboratory Standards Institute (CLSI) M100-S24 standards (MIC ug/ml ≤ 8 susceptible; 16 intermediate; ≥ 32 resistant).

### Association between NalD variation and aztreonam MIC

The ranksum statistic test measures if strains with high variable NalD protein sequence would have higher MIC than the strains with NalD similar to consensus sequence. NalD protein sequences from *P*. *aeruginosa* isolates are aligned and consensus sequence is obtained using Matlab bioinformatics toolbox. Protein variation is calculated by comparing each NalD to the consensus protein sequence built from the collection. A median value of the sequence variation was used as a cutoff to group the strains into high various and low various group. The MIC values in each group were then compared using ranksum test. Overall there is no cutoff drawn for the MIC value.

### Structural analysis of NalD

The 3D structure was obtained from protein data bank (PDB) [64] with ID 5daj [28]. The structure analysis was done in Pymol (The PyMOL Molecular Graphics System, Version 2.0 Schrödinger, LLC.) with educational-Use license.

### Statistical analysis and machine learning

All analyses were carried out using Matlab^™^ with the Statistics and Machine Learning toolbox. Aztreonam MIC and standard errors of different strains were estimated using function *fitlm* as:

$$\text{MIC}\sim 1+{\text{Var}}_{\text{strain}}$$

Wilcoxon rank sum test was performed using function *ranksum*. Antibiotic disk assay data were clustered using *seqlinkage* function based on pairwise distance (*pdist*). Elastic net regularization (in *lasso* function) was used to select for transcriptional regulators to predict antibiotic resistance with cross validation (*cvpartition*) and later to calculate the coefficient of selected transcriptional regulators using *fitlm*. For RNAseq analysis, we use the DESeq2 package to call the differentially expressed genes by p-value adjusted with multiple hypotheses (Benjamini-Hochberg method). For analyzing antibiotic disk diffusion, the diameter (D) of cleared zone caused by each antibiotic was measured using imageJ. The inhibition zone index (I) for each antibiotic was calculated across the strains as:

$$I=\frac{X-\overset{-}{X}}{S}$$

where S is the standard deviation of the data, X is the square of measured diameter D to represent the inhibition area.

Antibiotic resistance index (R) for strain j was calculated as: *R*_*j*_ = −Σ*I*_*i*_ where i refers to antibiotics used in disk diffusion assay.

### Integrative metabolic flux analysis

The boundary fluxes of the iJN1411 model were set to mimic environmental conditions in the experiment: the uptake fluxes of all 20 amino acids except tryptophan were set to 1 (in any unit) so all intracellular reactions have normalized fluxes relative to nutrient uptake.

To find the flux solution that has the highest consistency with gene expression data, we implemented the iMAT algorithm [65], which formulated a mixed integer linear programming (MILP) problem to maximize the total number of active reactions associated with highly expressed genes (denoted by *R*_*H*_) and inactive reactions associated with lowly expressed genes (denoted by *R*_*L*_) under a biomass constraint

$${\text{max}}_{v,y^{+},y^{-}}\sum_{i\in R_{H}}\left(y_{i}^{+}+y_{i}^{-}\right)+\sum_{i\in R_{L}}y_{i}^{+}$$ (1)

s.t.

S⋅v=0 (2)

$$v_{min}\leq v\leq v_{max}$$ (3)

$$v_{i}+y_{i}^{+}(v_{min,i}-\epsilon )\geq v_{min,i}\;\text{for}\;i\in R_{H}$$ (4)

$$v_{i}+y_{i}^{-}(v_{max,i}+\epsilon )\leq v_{max,i}\;\text{for}\;i\in R_{H}$$ (5)

$$v_{min,i}(1-y_{i}^{+})\leq v_{i}\leq v_{max,i}\text{(}1-y_{i}^{+})\;\text{for}\;i\in R_{L}$$ (6)

$$y_{i}^{+},y_{i}^{-}\in [0,1]$$

$$v_{bio}\geq f\cdot v_{max,bio}$$ (7)

*S* is the stoichiometric coefficient matrix of the iJN1411 model. *v* is a vector of metabolic flux, and *v*_*min*_ and *v*_*max*_ are their lower and upper bounds obtained by flux variability analysis. As suggested by [66], we used the top 25% and 75% gene expression thresholds to determine the set of lowly (<25% quantile) and highly (>75% quantile) expressed reactions. For reactions associated with multiple isozymes or one enzyme with multiple subunits, we determined their corresponding transcription levels by replacing “and” and “or” operators with “min” and “max” respectively in their gene-protein-reaction (GPR) rules. $y_{i}^{+}$ and $y_{i}^{-}$ are Boolean variables to indicate the flux activity of the reaction *i* in its forward and reverse direction respectively: highly expressed reactions are active if $y_{i}^{+}=1$ or $y_{i}^{-}=1$ and lowly expressed reactions are inactive if $y_{i}^{+}=1$. We chose *∊* = 0.1, which is a positive threshold for flux activity of highly expressed reactions: active reactions carry fluxes with absolute values equal or above *∊*. *v*_*bio*_ and *v*_*max*,*bio*_ are the biomass flux and its maximum possible value respectively, and *f* is a parameter that tunes the rigidity of the biomass constraint. We determined *f* = 0.95 from a trade-off analysis (S5 Fig), which chose a large value of *f* where the objective function remains near-optimal but starts to have diminishing returns by increasing *f* further.

The reference model for D-3rsw in the absence of aztreonam was constructed by constraining the reaction in the iJN1411 model using the iMAT solution (*v*_*iMAT*_). For any reaction *i*, we imposed the following constraints on its flux bounds: 0 ≤ *v*_*i*_ ≤ *v*_*iMAT*,*i*_ for *v*_*iMAT*,*i*_ > 0, −*v*_*iMAT*,*i*_ ≤ *v*_*i*_ ≤ 0 for *v*_*iMAT*,*i*_ < 0, and *v*_*i*_ = 0 for *v*_*iMAT*,*i*_ = 0. Metabolic models in other conditions were constructed by modifying the flux bounds of reactions in the reference model based on gene expression changes between these conditions and the reference condition, i.e., *v*_*min*,*i*_ → *v*_*min*,*i*_ · *c*_*i*_, *v*_*max*,*i*_ → *v*_*max*,*i*_ · *c*_*i*_, where *c*_*i*_ is the fold change in mRNA levels of genes associated with reaction *i*.

Custom Python codes were developed with the COBRApy package [67] to carry out all metabolic flux modeling and simulations in the paper. Flux variability analysis and flux sampling were performed using the built-in COBRApy function *flux_variability_analysis* and *sample* respectively.

## Supporting information

S1 Fig Aztreonam resistance is associated with NalD mutation. (A) NalD protein alignments ordered by the value of minimum inhibitory concentration (MIC) of aztreonam obtained for the corresponding isolate. None of the previously collected clinical isolates has the same mutation as in D+7bld NalD (F198L). (B) The protein sequence of NalD is highly conserved. The strains resistant to aztreonam tend to have mutations in NalD compared to the strains susceptible to aztreonam. The one that is most resistant to aztreonam, X9820 has a deletion of 134 amino acid residues at the beginning of NalD.
(TIF)

S2 Fig Growth curve synchronization method for precise measurement of growth rate of sepsis isolates D+5bld and D+7bld. The first column shows high-resolution growth curve of serially diluted cell inoculum. The middle column shows aligned curves on the left side. The last column on the right shows the determination of growth rate by linear fitting of time shift against dilution. There is no measurable cost to fitness *in vitro* for the strain carrying the aztreonam resistance mutation.
(TIF)

S3 Fig D+7bld shows no fitness cost measured in direct competition with D+5bld *in vitro* in the absence of aztreonam. Overnight competition between D+7bld and D+5bld cells in a 1:1000 initial rate is highly impacted by aztreonam concentration. When aztreonam is absent, D+7bld frequency remains unchanged after competition with D+5bld cells. At the sublethal aztreonam concentration of 2μg/mL D+7bld frequency increases, as expected, by ~10 fold. At aztreonam concentration of 4μg/mL, which is above the MIC of D+5bld but not D+7bld, D+7bld frequency increases more than 300 fold. (** p<0.01).
(TIF)

S4 Fig Variation in NalD and other transcriptional regulators could explain multiple drug resistance excluding aztreonam. The 13 transcriptional regulators overall explains >90% of the variation in the multi-drug resistance index calculated from 7 antibiotics (excluding aztreonam from Fig 4B). The coefficients have units of summarized fold-change across all the 7 antibiotics.
(TIF)

S5 Fig Trade-off between maximizing consistency between metabolic flux and gene expression and maximizing biomass production in the iMAT algorithm. The consistency score in the y axis is equal to the value of the objective function, which is given by Eq (1) in the main text. The parameter f in the x axis imposes a biomass constraint that requires the ratio of biomass flux to its maximum possible value is at least f. f = 0.95 is a point of diminishing return that increasing the minimum biomass flux return will lead to dramatic drop in the consistency score.
(TIF)

S1 Table Association between aztreonam resistance and protein sequence variation of the 19 proteins that have recurrent mutations during experimental evolution [13] (Ranksum test, total strain# = 31). (DOCX)

S2 Table Transcriptional regulators associated with aztreonam MIC. (DOCX)

S3 Table Mutations in the eight sepsis isolates are confirmed with Sanger sequencing. (DOCX)

S4 Table Primers used to generate mutations in PA14. (DOCX)

S5 Table Summary of small indels and SNPs comparing to PAO1. (DOCX)

S6 Table Blast result of the three big insertions. (DOCX)

S7 Table Transcriptional regulators associated with overall antibiotic resistance. (DOCX)

S1 Data Minimum inhibitory concentration of aztreonam for all clinical isolates. (XLSX)

S2 Data Experimental validation that NalD^F198L^ is associated with increase of aztreonam minimum inhibitory concentration. (XLSX)

S3 Data Inhibition zone measurement using antibiotic disk diffusion assay for clinical isolates. (XLSX)

S4 Data Genes change expression in the presence of aztreonam. (XLSX)

S5 Data Constitutive mutation effects. Forty-eight reactions constitutively affected by mutations in D+7bld regardless of the presence and concentration of aztreonam.
(XLSX)

S1 Text Supplementary materials and methods. (DOCX)
