# Supplementary figures and images for: Systems-level analysis of NalD mutation, a recurrent driver of rapid drug resistance in acute Pseudomonas aeruginosa infection
Source: PLoS Comput Biol. 2019 Dec 20;15(12):e1007562. doi: 10.1371/journal.pcbi.1007562 (PMC6944390; doi:10.1371/journal.pcbi.1007562)

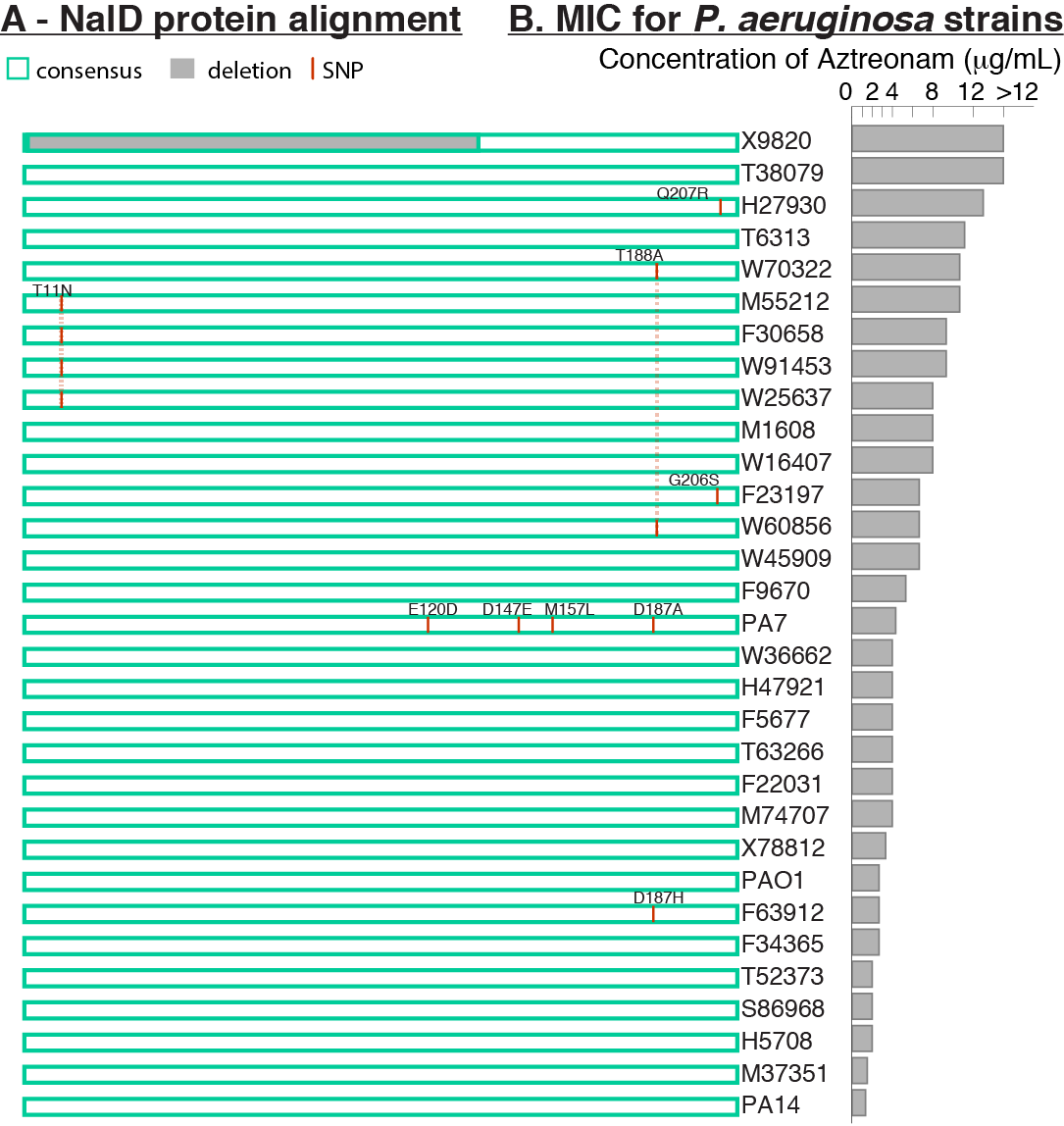

Supplement: S1 Fig — (A) NalD protein alignments ordered by the value of minimum inhibitory concentration (MIC) of aztreonam obtained for the corresponding isolate. None of the previously collected clinical isolates has the same mutation as in D+7bld NalD (F198L). (B) The protein sequence of NalD is highly conserved. The strains resistant to aztreonam tend to have mutations in NalD compared to the strains susceptible to aztreonam. The one that is most resistant to aztreonam, X9820 has a deletion of 134 amino acid residues at the beginning of NalD. (TIF) [file pcbi.1007562.s001.tif]

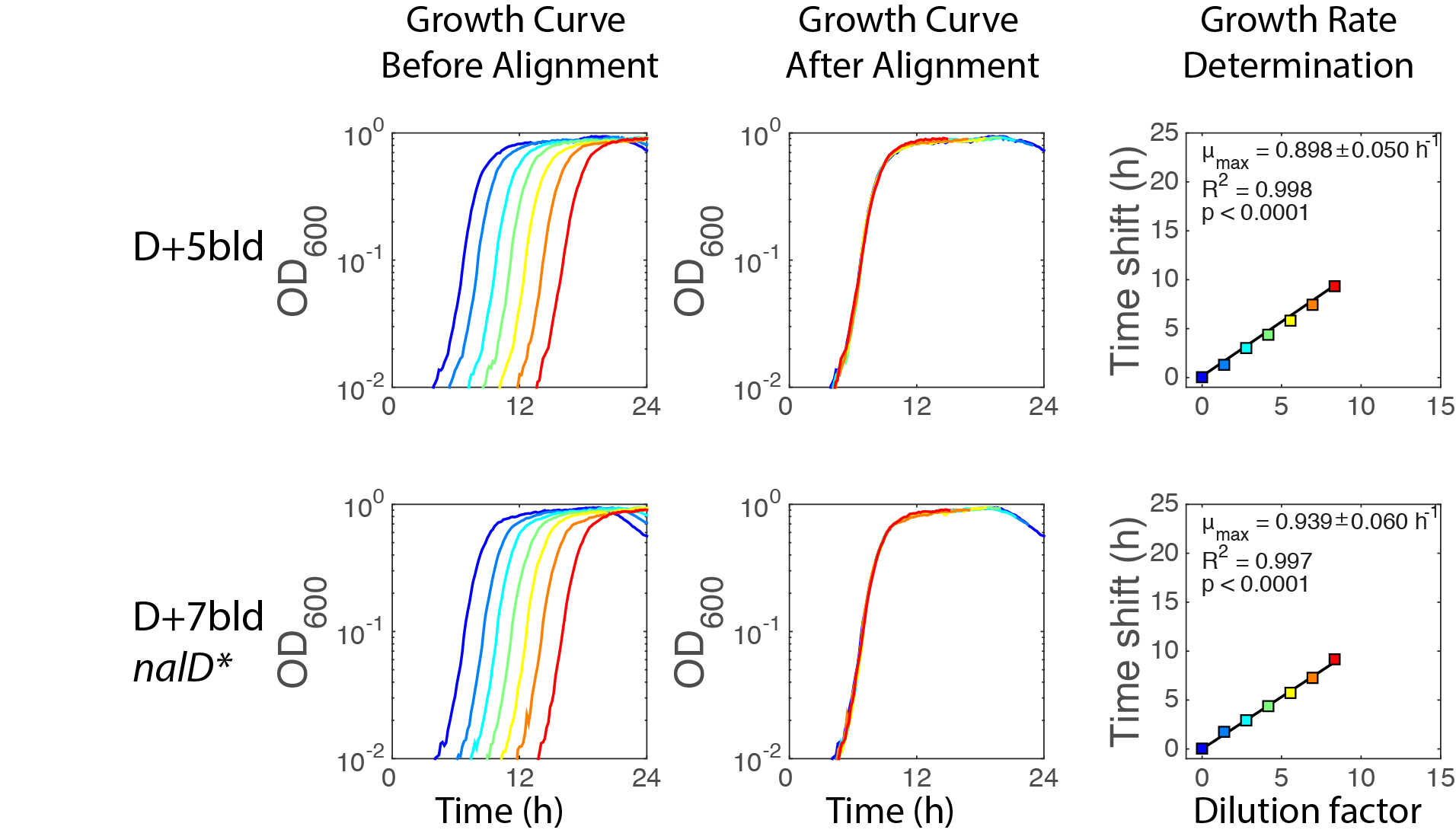

Supplement: S2 Fig — The first column shows high-resolution growth curve of serially diluted cell inoculum. The middle column shows aligned curves on the left side. The last column on the right shows the determination of growth rate by linear fitting of time shift against dilution. There is no measurable cost to fitness in vitro for the strain carrying the aztreonam resistance mutation. (TIF) [file pcbi.1007562.s002.tif]

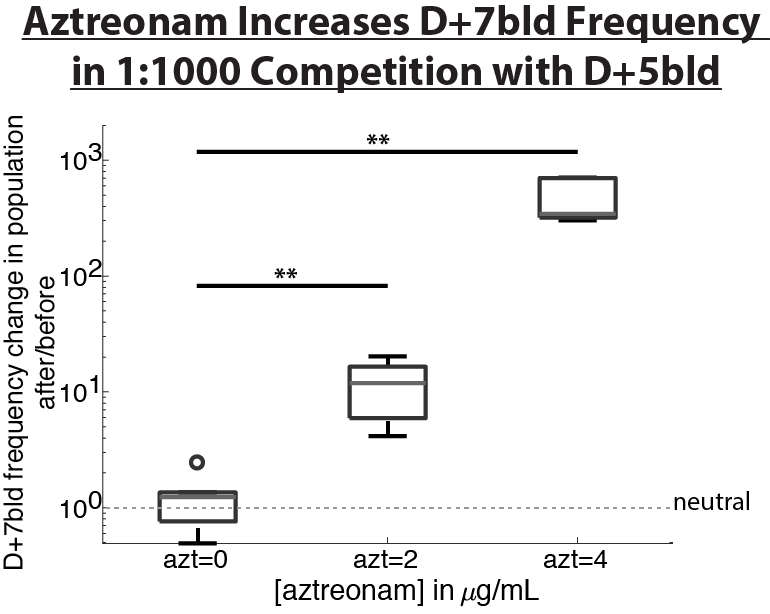

Supplement: S3 Fig — Overnight competition between D+7bld and D+5bld cells in a 1:1000 initial rate is highly impacted by aztreonam concentration. When aztreonam is absent, D+7bld frequency remains unchanged after competition with D+5bld cells. At the sublethal aztreonam concentration of 2μg/mL D+7bld frequency increases, as expected, by ~10 fold. At aztreonam concentration of 4μg/mL, which is above the MIC of D+5bld but not D+7bld, D+7bld frequency increases more than 300 fold. (** p<0.01). (TIF) [file pcbi.1007562.s003.tif]

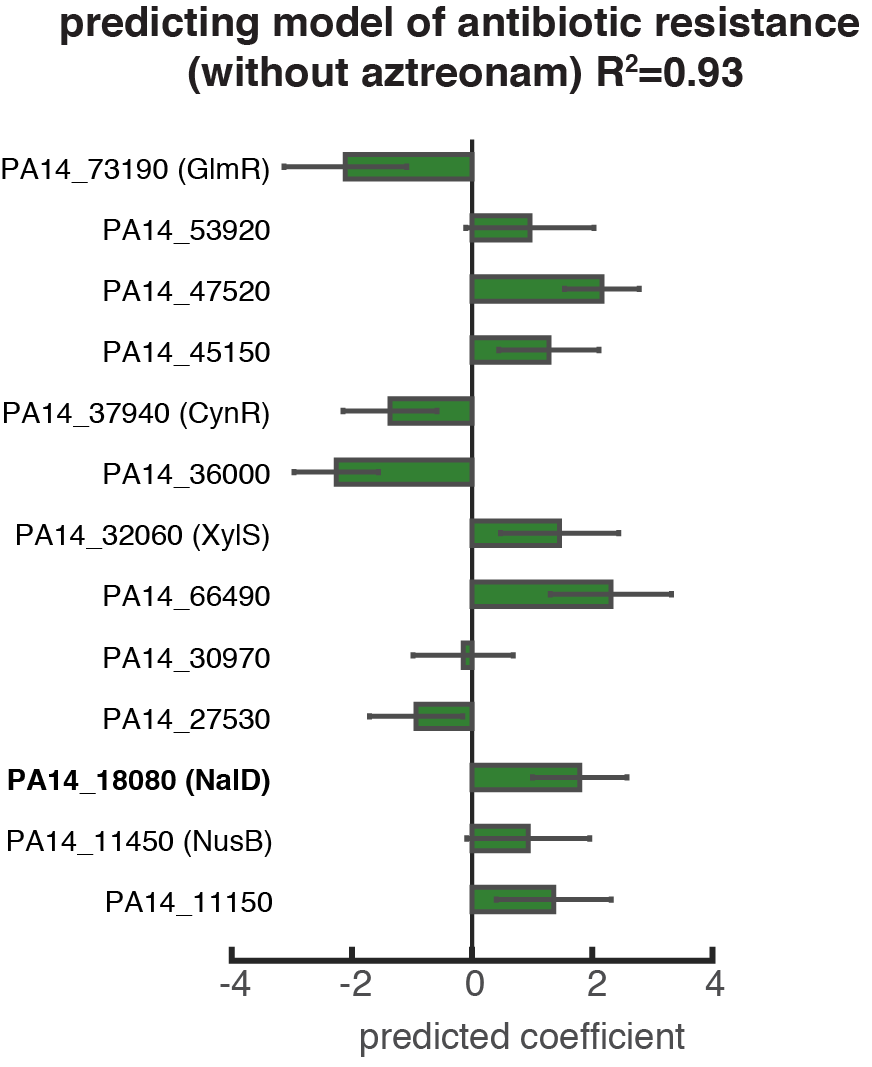

Supplement: S4 Fig — The 13 transcriptional regulators overall explains >90% of the variation in the multi-drug resistance index calculated from 7 antibiotics (excluding aztreonam from Fig 4B). The coefficients have units of summarized fold-change across all the 7 antibiotics. (TIF) [file pcbi.1007562.s004.tif]

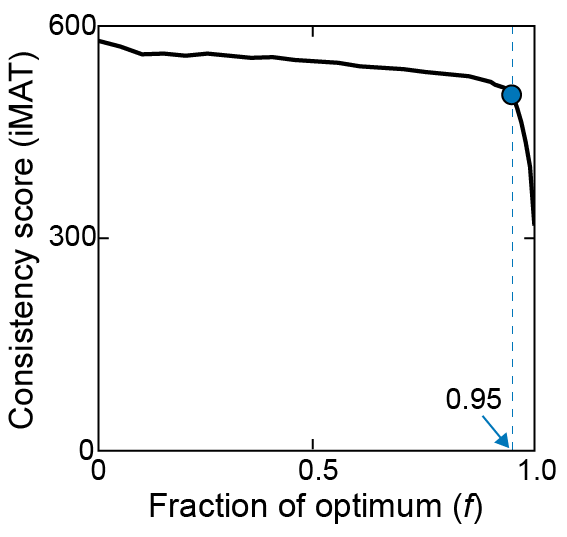

Supplement: S5 Fig — The consistency score in the y axis is equal to the value of the objective function, which is given by Eq (1) in the main text. The parameter f in the x axis imposes a biomass constraint that requires the ratio of biomass flux to its maximum possible value is at least f. f = 0.95 is a point of diminishing return that increasing the minimum biomass flux return will lead to dramatic drop in the consistency score. (TIF) [file pcbi.1007562.s005.tif]
