# Supplementary material for: Systems-level analysis of NalD mutation, a recurrent driver of rapid drug resistance in acute Pseudomonas aeruginosa infection
Source: PLoS Comput Biol. 2019 Dec 20;15(12):e1007562. doi: 10.1371/journal.pcbi.1007562 (PMC6944390; doi:10.1371/journal.pcbi.1007562)
Supplement: S1 Table — (DOCX) [file pcbi.1007562.s006.docx]

**Supplementary Table 1** **Association between aztreonam resistance and protein sequence variation of the 19 proteins that have recurrent mutations during experimental evolution** [1] (Ranksum test, total strain# = 31).

| **geneName** | **locus** | **pvalue** |
| --- | --- | --- |
| mexR | PA0424 | 0.76 |
| ftsI | PA4418 | 0.41 |
| phoQ | PA1180 | 0.51 |
| mexF | PA2494 | 0.91 |
| aroB | PA5038 | 0.21 |
| mpl | PA4020 | 0.07 |
| clpA | PA2620 | 0.47 |
| mexT | PA2492 | 0.93 |
| orfN | PA14_23460 | 0.054 |
| pgi | PA4732 | 1 |
| clpS | PA2621 | 0.93 |
| PA3206 |  | 0.11 |
| dacB | PA3047 | 0.29 |
| pepA | PA3831 | 0.18 |
| ampC | PA4110 | 0.06 |
| atpA | PA5556 | 0.16 |
| atpD | PA5554 | no sequence variation |
| nalD | PA3574 | **0.005** |
| nalC | PA3721 | 0.54 |

**Reference**

1. Jorth P, McLean K, Ratjen A, Secor PR, Bautista GE, et al. (2017) Evolved Aztreonam Resistance Is Multifactorial and Can Produce Hypervirulence in *Pseudomonas aeruginosa.* MBio 8. doi:10.1128/mBio.00517-17.
