# Supplementary material for: Systems-level analysis of NalD mutation, a recurrent driver of rapid drug resistance in acute Pseudomonas aeruginosa infection
Source: PLoS Comput Biol. 2019 Dec 20;15(12):e1007562. doi: 10.1371/journal.pcbi.1007562 (PMC6944390; doi:10.1371/journal.pcbi.1007562)
Supplement: S2 Table — (DOCX) [file pcbi.1007562.s007.docx]

**Supplementary Table 2 transcriptional regulators associated with aztreonam MIC**

| **Locus tag in bld+7** | **Locus tag in PA14** | **Locus tag in PAO1** | **Gene name** | **Function** | **References** |
| --- | --- | --- | --- | --- | --- |
| 287.2839.peg.1518 | PA14_18080 | PA3574 | nalD |  | [1,2] |
| 287.2839.peg.1983 | PA14_23700 | PA3124 |  |  |  |
| 287.2839.peg.2369 | PA14_63170 | PA4778 | *cueR* | Regulated by LasR; regulate mexPQ-opmE | [3] |
| 287.2839.peg.4139 | PA14_43770 | PA1603 |  |  |  |
| 287.2839.peg.3105 | PA14_33170 | PA2432 | *bexR* | LysR family, controls virulence gene expression | [4] |
| 287.2839.peg.882 | PA14_10290 | PA4147 | *acoR* |  |  |
| 287.2839.peg.341 | PA14_03840 | PA0294 | *aguR* | TetR family |  |
| 287.2839.peg.3771 | PA14_40440 | PA1859 |  |  |  |
| 287.2839.peg.2984 | PA14_31560 | PA2551 |  |  |  |
| 287.2839.peg.5911 | PA14_65900 | PA4984 |  |  |  |
| 287.2839.peg.5828 | PA14_64820 | PA4906 |  |  |  |
| 287.2839.peg.3390 | PA14_36000 | PA2005 | *hbcR* | (R)-3-hydroxybutyrate catabolism regulator | [5] |
| 287.2839.peg.4618 | PA14_49630 | PA1142 |  |  |  |
| 287.2839.peg.561 | PA14_06400 | PA0491 |  | LysR family |  |
| 287.2839.peg.3237 | PA14_34690 | PA2316 |  | LysR family |  |
| 287.2839.peg.2553 | PA14_28420 | PA2758 |  | LysR family |  |
| 287.2839.peg.549 | PA14_06260 | PA0479 |  | LysR family |  |
| 287.2839.peg.4624 | PA14_49700 | PA1136 |  |  |  |
| 287.2839.peg.1457 | PA14_17380 | PA3630 | *gfnR* | glutathione-dependent formaldehyde neutralization regulator | [6] |
| 287.2839.peg.6578 | PA14_72890 | PA5525 |  |  |  |
| 287.2839.peg.480 | PA14_05420 | PA0416 |  |  |  |
| 287.2839.peg.4366 | PA14_46400 | PA1380 |  |  |  |
| 287.2839.peg.4339 | PA14_46170 | PA1413 |  |  |  |
| 287.2839.peg.4472 | PA14_47610 | PA1283 |  |  |  |
| 287.2839.peg.547 | PA14_06240 | PA0477 |  |  |  |
| 287.2839.peg.3490 | PA14_37120 | PA2123 |  |  |  |
| 287.2839.peg.5491 | PA14_60860 | PA4600 | *nfxB* | resistance to quinolones , repressor of efflux pump mexCD-oprJ and itself | [7,8] |
| 287.2839.peg.4416 | PA14_46990 | PA1335 | *aauR* | acidic amino acid uptake | [9] |
| 287.2839.peg.636 | PA14_07340 | PA0564 |  |  |  |
| 287.2839.peg.4465 | PA14_47520 | PA1290 |  |  |  |

**References**

1. Chen W, Wang D, Zhou W, Sang H, Liu X, et al. (2016) Novobiocin binding to NalD induces the expression of the MexAB-OprM pump in *Pseudomonas aeruginosa*. Mol Microbiol 100: 749–758. doi:10.1111/mmi.13346.

2. Sobel ML, Hocquet D, Cao L, Plesiat P, Poole K (2005) Mutations in PA3574 (nalD) lead to increased MexAB-OprM expression and multidrug resistance in laboratory and clinical isolates of *Pseudomonas aeruginosa*. Antimicrob Agents Chemother 49: 1782–1786. doi:10.1128/AAC.49.5.1782-1786.2005.

3. Thaden JT, Lory S, Gardner TS (2010) Quorum-sensing regulation of a copper toxicity system in *Pseudomonas aeruginosa.* J Bacteriol 192: 2557–2568. doi:10.1128/JB.01528-09.

4. Turner KH, Vallet-Gely I, Dove SL (2009) Epigenetic control of virulence gene expression in *Pseudomonas aeruginosa* by a LysR-type transcription regulator. PLoS Genet 5: e1000779. doi:10.1371/journal.pgen.1000779.

5. Lundgren BR, Harris JR, Sarwar Z, Scheel RA, Nomura CT (2015) The metabolism of (R)-3-hydroxybutyrate is regulated by the enhancer-binding protein PA2005 and the alternative sigma factor RpoN in *Pseudomonas aeruginosa* PAO1. Microbiology (Reading, Engl) 161: 2232–2242. doi:10.1099/mic.0.000163.

6. Willsey GG, Wargo MJ (2016) Sarcosine Catabolism in *Pseudomonas aeruginosa* Is Transcriptionally Regulated by SouR. J Bacteriol 198: 301–310. doi:10.1128/JB.00739-15.

7. Shiba T, Ishiguro K, Takemoto N, Koibuchi H, Sugimoto K (1995) Purification and characterization of the *Pseudomonas aeruginosa* NfxB protein, the negative regulator of the nfxB gene. J Bacteriol 177: 5872–5877.

8. Okazaki T, Hirai K (1992) Cloning and nucleotide sequence of the *Pseudomonas aeruginosa* *nfxB* gene, conferring resistance to new quinolones. FEMS Microbiol Lett 76: 197–202.

9. Singh B, Röhm K-H (2008) Characterization of a *Pseudomonas putida* ABC transporter (AatJMQP) required for acidic amino acid uptake: biochemical properties and regulation by the Aau two-component system. Microbiology (Reading, Engl) 154: 797–809. doi:10.1099/mic.0.2007/013185-0.
