# Supplementary material for: Systems-level analysis of NalD mutation, a recurrent driver of rapid drug resistance in acute Pseudomonas aeruginosa infection
Source: PLoS Comput Biol. 2019 Dec 20;15(12):e1007562. doi: 10.1371/journal.pcbi.1007562 (PMC6944390; doi:10.1371/journal.pcbi.1007562)
Supplement: S3 Table — (DOCX) [file pcbi.1007562.s008.docx]

**Supplementary Table 3. mutations in the eight sepsis isolates are confirmed with Sanger Sequencing**

| Mutation identified by WGS | strain | PCR primers | Confirmed  by Sanger  Sequencing |
| --- | --- | --- | --- |
| *pslI _*1854A-> C | D+3bld | Fw: GTAGGACGGTTGCCAGAGC  Rv: ACATCGACCTGTACATCAGCAC | Yes |
| *peg.1088*_467  C-> A | D+4rsw | Fw: ACTGGTCGTAGGCCTGGTC  Rv: GTCGTTGAACGTCGGCTACTAC | Yes |
| *peg.6349*_1036  G->A | D+7spt | Fw: AACTCGATCACCGAGAGCAC  Rv: GCCAAGCAACTGATGGTCTATG | Yes |
| *peg.6504*_900  A-> G | D+4rsw | Fw: CAGTTTTCCGCCTCAAGATG  Rv: AAGGTGGAGTACCTGCCCTAC | Yes |
| *nalD*592T->C | D+7bld | Fw: CATGCAAGTCTTCAAGGTTCAG  Rv: CTGACCATCCTGATGCAACG | Yes |
| *peg.4653* Δ333-342 | D+7bld | Fw: CAGCTTGCGTTTCGTCAGTC  Rv: ACGATCTTGCCCTGCAACTT | Yes |
| 2095237Δ191bp | D+4rsw | Fw: TCAGGGCATTGACTTAGGTTTT  Rv: TCAGCTGTAGACGAGGAAGATG | Yes |
| 6349089 G->A | D+4rsw | Fw: ACCCAGTTGACATAAGCCTGTT  Rv: GCGTTTGTGGATACATCGTAAA | No (sequencing error) |
| *czcD*Δ484-486 | D+3bld | Fw: GGGCTGATGCTCTACCAATCG  Rv: GGTGATGTGCACCAGTTCCTC | No (sequencing error) |
