# Supplementary material for: Systems-level analysis of NalD mutation, a recurrent driver of rapid drug resistance in acute Pseudomonas aeruginosa infection
Source: PLoS Comput Biol. 2019 Dec 20;15(12):e1007562. doi: 10.1371/journal.pcbi.1007562 (PMC6944390; doi:10.1371/journal.pcbi.1007562)
Supplement: S4 Table — (DOCX) [file pcbi.1007562.s009.docx]

**Supplementary Table 4. Primers used to generate mutations in PA14**

| Target gene | Primer names | Primer sequences (5’->3’) | Purposes and resources |
| --- | --- | --- | --- |
| PA14_50010 (peg.4653) | 4563FW | *GGG GAC AAG TTT GTA CAA AAA AGC AGG CTC A* GCTTGCGTTTCGTCAGTCC | For allelic exchange of the 10bp deletion into PA14 genome. This study |
|  | 4563RV | *GGG GAC CAC TTT GTA CAA GAA AGC TGG GTA* AAGGGATTGTCCTTGGGAACG |  |
| PA14_18080 (nalD) | nalD_IF_upFw | *GGG GAC AAG TTT GTA CAA AAA AGC AGG CTC A* GCCGTCTACTGGCACTTCCAG | For in-frame deletion of the 45bp nucleotides that code for the 10th α-helix of NalD protein and engineering *NalD^F198L^* SNP into PA14. |
|  | nalD_IF_upRv | *CTG ACC CCA GTC GCG CAC CAG*  ATC GAA CAG GCG CGG GTC | For in-frame deletion of the 45bp nucleotides that code for the 10th α-helix of NalD protein |
|  | nalD_IF_downFw | CTG GTG CGC GAC TGG GG | For in-frame deletion of the 45bp nucleotides that code for the 10th α-helix of NalD protein and engineering *NalD^F198L^* SNP into PA14. |
|  | nalD_IF_downRv | *GGG GAC CAC TTT GTA CAA GAA AGC TGG GTA* GCA GGG CGA TGA TCA GCA GG | For in-frame deletion of the 45bp nucleotides that code for the 10th α-helix of NalD protein and engineering *NalD^F198L^* SNP into PA14. |
| PA14_18080 (nalD) | *nalD_snp_upRV* | *CTG ACC CCA GTC GCG CAC CAG*  GCC ACG GA***G*** CAT CGG CTC C | For engineering *NalD^F198L^* SNP into PA14. *The rest primers are the same as used in in-frame deletion. |
| PA14_18080 (nalD) | nalD_IF_seqFW | AGCAGCATTAGACAAAGGTGGTGTCG | To amplify and confirm the *NalDΔα10* and *NalD^F198L^* construction in PA14 using Sanger Sequencing |
|  | nalD_IF_seqRV | AGACGTTGCCCTTGCCGC |  |
