# Supplementary material for: Systems-level analysis of NalD mutation, a recurrent driver of rapid drug resistance in acute Pseudomonas aeruginosa infection
Source: PLoS Comput Biol. 2019 Dec 20;15(12):e1007562. doi: 10.1371/journal.pcbi.1007562 (PMC6944390; doi:10.1371/journal.pcbi.1007562)
Supplement: S5 Table — (DOCX) [file pcbi.1007562.s010.docx]

**Supplementary Table 5.** Summary of small indels and SNPs comparing to PAO1

| **Mutation in sepsis isolates** | **Location in PAO1** | **function** | **KEGG pathway** | **Present in sepsis isolate(s)** |
| --- | --- | --- | --- | --- |
| *oprD*^Δ^*^1336bp^* | oprD (PA0958) | Outer memberane porin | beta-Lactam resistance | D+4rsw |
| D+4rsw^Δ191bp^ | intergenic |  |  | D+4rsw |
| Δ1635bp | intergenic |  |  | D-3rsw  D+4rsw  D+3bld  D+4bld  D+5bld  D+7bld |
| *D+7bld.peg.4653^Δ10bp^* | PA1112 | dehydrogenase |  | D+7bld |
| *nalD^T592C^(F198L)* | PA3574 | negative regulation of transmembrane transport | beta-Lactam resistance | D+7bld |
| *pslI^T618G^ (syn)* | PA2239 | extracellular polysaccharide biosynthesis | Biofilm formation | D-10rsw  D-3rsw  D+4rsw  D+4bld  D+5bld  D+7bld  D+7spt |
| *D-10rsw.peg.1083^A467C^(D156A)* | PA3959 | hypothetical protein |  | D-10rsw  D-3rsw  D+3bld  D+4bld  D+5bld  D+7bld  D+7spt |
| *pauC^C1026T^(syn)* | PA5312 | Aldehyde dehydrogenase | Arginine and proline metabolism | D+7spt |
| *D-10rsw.peg.6594^G900A^* | PA5545 |  | conserved hypothetical protein, TRAP transporter solute receptor, TAXI family | D-10rsw  D-3rsw  D+4bld  D+5bld  D+7bld  D+7spt |
