# Supplementary material for: Systems-level analysis of NalD mutation, a recurrent driver of rapid drug resistance in acute Pseudomonas aeruginosa infection
Source: PLoS Comput Biol. 2019 Dec 20;15(12):e1007562. doi: 10.1371/journal.pcbi.1007562 (PMC6944390; doi:10.1371/journal.pcbi.1007562)
Supplement: S6 Table — (DOCX) [file pcbi.1007562.s011.docx]

**Supplementary Table 6.** Blast result of the three big insertions

| Ins size & strain | Top hit | features | target sequence | Query coverage |
| --- | --- | --- | --- | --- |
| 10716 bp  D+4bld | Genomic repeat of *D+4bld*  *1351097-1358225; 1361811-1368939* | peg.1297  "hypothetical protein"    peg.1298  " O-antigen acetylase"    peg.1299  "putative oxidoreductase"    peg.1300  “Short-chain dehydrogenase/reductase SDR“    peg.1301  “hypothetical protein”    peg.1302  “Transcriptional regulator, AraC family“    peg.1303  “hypothetical protein”    peg.1304  “Putative oxidoreductase YncB”    peg.1305  Multicopper oxidase    Peg.1306  tRNA-specific adenosine-34 deaminase (EC 3.5.4.33)    peg.1307  “hypothetical protein”    peg.1308  “probable aromatic amino acid transporter | direct repeat of self genome |  |
| Δ61827 bp  D-4rsw | *P. aeruginosa* strain E6130952 CP020603.1  3904538: 3912719 | 3904569..3904916 "tail protein"    3904917..3908306  "phage tail length tape measure protein"    3909223..3909489  "hypothetical protein"    3909519..3909869  "hypothetical protein"    3909879..3910382  "hypothetical protein"    3910446..3910811  "hypothetical protein"    3910811..3911284  "phage-like protein"    3911277..3911894  "hypothetical protein"    3911845..3912657  "hypothetical protein" | Alignment of top hit in NCBI  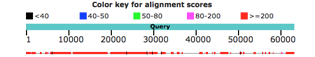 | 62% |
| 14064 bp  D+4rsw | *Pseudomonas aeruginosa* strain PA83  CP017293.1    6206183 to 6218768 | 6207276..6207914  "TniR protein"    6207961..6209178  "TniQ protein"    6209175..6210083  TniB NTP-binding protein    6210086..6211801  "TniA transposase"    6211937..6212926  diguanylate phosphodiesterase    6212923..6213159  "putative mercury resistance protein"    6213156..6213521  "transcriptional regulator MerD"    6213539..6215224  "putative mercuric reductase"    6215296..6215571  "putative periplasmic mercuric ion binding protein"    6215587..6215937  "putative mercuric transport protein"    6216009..6216443  "putative transcriptional regulator MerR"    6216471..6217322  "Resolvase domain protein-containing protein"    6217613..6218626  "integrase/recombinase" | Alignment of top hit in NCBI  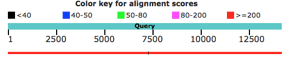 | 99% |
