# Supplementary material for: Systems-level analysis of NalD mutation, a recurrent driver of rapid drug resistance in acute Pseudomonas aeruginosa infection
Source: PLoS Comput Biol. 2019 Dec 20;15(12):e1007562. doi: 10.1371/journal.pcbi.1007562 (PMC6944390; doi:10.1371/journal.pcbi.1007562)
Supplement: S7 Table — (DOCX) [file pcbi.1007562.s012.docx]

**Supplementary Table 7.** Transcriptional regulators associated with overall antibiotic resistance.

| Locus tag in bld+7 | Locus tag in PA14 | Locus tag in PAO1 | Gene name | Function | References |
| --- | --- | --- | --- | --- | --- |
| Peg.6604 | PA14_73190 | PA5550 | *glmR* |  |  |
| peg.4983 | PA14_53920 | PA0797 |  |  |  |
| Peg.3563 | PA14_37940 | PA2054 | *cynR* |  |  |
| peg.3390 | PA14_36000 | PA2005 | hbcR | (R)-3-hydroxybutyrate catabolism regulator | [1] |
| peg.1518 | PA14_66490 |  | *nalD* |  | [2,3] |

**References**

1. Lundgren BR, Harris JR, Sarwar Z, Scheel RA, Nomura CT (2015) The metabolism of (R)-3-hydroxybutyrate is regulated by the enhancer-binding protein PA2005 and the alternative sigma factor RpoN in *Pseudomonas aeruginosa* PAO1. Microbiology (Reading, Engl) 161: 2232–2242. doi:10.1099/mic.0.000163.

2. Chen W, Wang D, Zhou W, Sang H, Liu X, et al. (2016) Novobiocin binding to NalD induces the expression of the MexAB-OprM pump in *Pseudomonas aeruginosa*. Mol Microbiol 100: 749–758. doi:10.1111/mmi.13346.

3. Sobel ML, Hocquet D, Cao L, Plesiat P, Poole K (2005) Mutations in PA3574 (nalD) lead to increased MexAB-OprM expression and multidrug resistance in laboratory and clinical isolates of *Pseudomonas aeruginosa*. Antimicrob Agents Chemother 49: 1782–1786. doi:10.1128/AAC.49.5.1782-1786.2005.
