# Supplementary material for: Systems-level analysis of NalD mutation, a recurrent driver of rapid drug resistance in acute Pseudomonas aeruginosa infection
Source: PLoS Comput Biol. 2019 Dec 20;15(12):e1007562. doi: 10.1371/journal.pcbi.1007562 (PMC6944390; doi:10.1371/journal.pcbi.1007562)
Supplement: S1 Text — (DOCX) [file pcbi.1007562.s018.docx]

# Supplementary Information

## **Detailed Materials and methods**

**Strains, genomes and laboratory culture.** All strains were grown overnight in lysogeny broth (LB) at 37°C with shaking at 250 rpm. Growth assays used casamino acid media prepared with 5g/L casamino acid (Bacto), 1 mM MgSO_4_, 0.1 mM CaCl_2_ and 1X buffer (12 g/L Na_2_HPO_4_ (Fisher Scientific), 15 g/L KH_2_PO_4_ (Fisher Scientific) and 2.5 g/L NaCl, pH6.7) [1]. Agar plates contain 1.5% agar (Bacto). Growth rate of D+7bld and D-3rsw in Figure S2 is measured by growth curve synchronization method in casamino acid [2].

**Antibiotic resistance assays.** We used a standard filter paper disk diffusion assay [3] to quantify the antibiotic sensitivities of *P. aeruginosa* strains. *P. aeruginosa* strains were inoculated from glycerol stock to LB and grown overnight at 37°C with shaking at 250 rpm. Cells of each strain was spread on LB agar plate with sterile cotton tipped applicator and let dry. Sterile filter paper disks were then placed onto the agar with different amount of antibiotics (Ciprofloxacin-1µg, Gentamicin-10µg, Aztreonam-32µg, Chloramphenicol-60µg, Ampicillin-200µg, Tetracycline-25µg, Meropenem-10µg, Cefepime-30µg). After overnight incubation at 37°C, the plates were imaged and the diameter of inhibition zone was measured with Fiji distributor of imageJ [4]**.**

The aztreonam minimum inhibitory concentration (MIC) assay was based on a standard protocol [5]. Overnight *P. aeruginosa* cultures were washed with PBS, diluted to final OD_600_ = 0.0025 in fresh casamino acid media with different concentration of aztreonam (0, 0.5, 1, 2, 4, 8, 12 μg/ml), and monitored with TECAN Infinite M1000 or Infinite M1000 Pro plate reader (Männedorf, Switzerland). An MIC was determined when the OD_600_ absorbance is less than 0.06 in TECAN after 24h incubation at 37°C. Assay was repeated three times, each with three technical replicates.

**Genomic sequencing and bioinformatics analysis.** The genomes of eight *P. aeruginosa* isolates were extracted using Genomic DNA Buffer Set and Genomic tip (Qiagen) and sequenced using PacBio as previously described [6]. Assembled genomes were then cleaned and circularized using in house tools. The mutations were identified by aligning genomes using mugsy [7]. Only mutations that were not single nucleotide insertion/gap in a poly-N area were included. Sanger sequencing confirmed mutations of single nucleotide polymorphism and small indels (Table S1)**.** The phylogenetic tree of *P. aeruginosa* clinical isolates was built using hamming distance and neighbor joining method as described [6]. The genomes are annotated in PATRIC database [8,9].

**Mutagenesis in laboratory strain PA14.** Both mutants *NalDΔα10* and *NalD^F198L^* were constructed as previously described [6] following the two-step allelic exchange prototal [10]. Primers used are listed in Supplementary Table 2. Briefly, two ~500 bp sequences upstream and downstream of targeted *nalD* mutation was amplified from laboratory strain PA14. For in-frame deletion of the 10th alpha helix of NalD, an extra splicing overlap extension (SOE) step was performed prior to ligation to plasmid, which eliminated a 45 bp fragment that codes for the 10^th^ alpha helix. To construct the point mutation from D+7bld into PA14 and avoid other non-synonymous mutation, the point mutation was introduced in the SOE primer step but no sequence was deleted. The fragment was used for allelic exchange to substitute the *nalD* sequence in *P. aeruginosa* laboratory strain PA14. A suicide plasmid introduced the amplified allele into targeted *P. aeruginosa* genome through homologous recombination [11]. One copy of the sequence was excised out by sucrose counter selection and the mutated clones were confirmed by Sanger sequencing (Genewiz Inc.).

**Competition assay between azt^s^ and azt^r^ strains**

To test the frequency change, overnight culture of D+7bld and D+5bld were harvested, washed with PBS, normalized to OD_600_=1 and mixed at a ratio of 1:1000. For competition, cell mixture was diluted to OD_600_=0.0025 in fresh LB with 0, 2, or 4μg/mL of aztreonam and grew overnight. Cell concentration was tested as colony forming unit (CFU) on casamino acids agar plates with (D+7bld) or without (mix: D+7bld& +5bld) 4μg/mL aztreonam. The fold change of D+7bld frequency was calculated as:

([D+7bld] / [mix])_after_ / ([D+7bld] / [mix])_before_

Data were collected from two experiments of three replicates. Statistical analysis was carried out using Matlab (R2017a, The MathWorks, Inc) function fitlm.

**Transcriptome sequencing and analysis.** Bacterial cells were inoculated from glycerol stock and incubated at 37°C overnight with shaking at 250rpm. The cells were diluted 1:600 in fresh LB with or without aztreonam the next day and brought back to mid-exponential growth. Total RNA was extracted using TRIzol^TM^ Reagent (Invitrogen) and treated with DNaseI to remove residuals of DNA. RNeasy columns (Qiagen) were used to further clean up the RNA. Libraries were prepared using Ribo-Zero^TM^ Gram Negative Bacteria Kit (Illumina ) and TruSeq stranded mRNA kit (Illumina), and loaded into MiSeq^®^ Reagent Kit v3 (600 cycle, Illumina) or MiSeq^®^ Reagent Kit v2 (500 cycle, Illumina) to be sequenced in MiSeq (Illumina).

The fastq files of our transcriptomic assay were filtered using fastQC (https://www.bioinformatics.babraham.ac.uk/projects/fastqc/). Possible adapter sequences were removed using Trimmomatic [12]. Gene counts and alignments were obtained by STAR [13] and SeQC [14]. Differentially expressed genes were identified using R package DESEQ2 [15].

The function category of differentially expressed genes were identified using eggNOG [16].

**Identification of transcriptional regulators**. All annotated genomes of *P. aeruginosa* are available through the National Center for Biotechnology Information (NCBI) and Patric [8]. The query transcriptional regulators were extracted from the sepsis isolate D+7bld and orthologous coding sequences for each transcriptional regulator were identified using Matlab function blastlocal against each genome. Any transcriptional regulators that have no ortholog in >=10 genomes were removed from the analysis (ortholog with e-value<1e-10). Each transcriptional regulator’s variation comparing to consensus sequence was then calculated and the median value was used as a cutoff to group the transcriptional regulators into ‘high variation’ and ‘low variation’ categories. To achieve a better statistical test power, if any group contains less than 5 isolates, the corresponding transcriptional regulator is also removed from the analysis.

**References**

1. Xavier JB, Kim W, Foster KR (2011) A molecular mechanism that stabilizes cooperative secretions in *Pseudomonas aeruginosa*. Mol Microbiol 79: 166–179. doi:10.1111/j.1365-2958.2010.07436.x.

2. van Ditmarsch D, Xavier JB (2011) High-resolution time series of *Pseudomonas aeruginosa* gene expression and rhamnolipid secretion through growth curve synchronization. BMC Microbiol 11: 140. doi:10.1186/1471-2180-11-140.

3. De Beer EJ, Sherwood MB (1945) The Paper-Disc Agar-Plate Method for the Assay of Antibiotic Substances. J Bacteriol 50: 459–467.

4. Schindelin J, Arganda-Carreras I, Frise E, Kaynig V, Longair M, et al. (2012) Fiji: an open-source platform for biological-image analysis. Nat Methods 9: 676–682. doi:10.1038/nmeth.2019.

5. Andrews JM (2001) Determination of minimum inhibitory concentrations. J Antimicrob Chemother 48 Suppl 1: 5–16. doi:10.1093/jac/48.suppl_1.5.

6. Yan J, Deforet M, Boyle KE, Rahman R, Liang R, et al. (2017) Bow-tie signaling in c-di-GMP: Machine learning in a simple biochemical network. PLoS Comput Biol 13: e1005677. doi:10.1371/journal.pcbi.1005677.

7. Angiuoli SV, Salzberg SL (2011) Mugsy: fast multiple alignment of closely related whole genomes. Bioinformatics 27: 334–342. doi:10.1093/bioinformatics/btq665.

8. Wattam AR, Abraham D, Dalay O, Disz TL, Driscoll T, et al. (2014) PATRIC, the bacterial bioinformatics database and analysis resource. Nucleic Acids Res 42: D581-91. doi:10.1093/nar/gkt1099.

9. Wattam AR, Davis JJ, Assaf R, Boisvert S, Brettin T, et al. (2017) Improvements to PATRIC, the all-bacterial Bioinformatics Database and Analysis Resource Center. Nucleic Acids Res 45: D535–D542. doi:10.1093/nar/gkw1017.

10. Hmelo LR, Borlee BR, Almblad H, Love ME, Randall TE, et al. (2015) Precision-engineering the *Pseudomonas aeruginosa* genome with two-step allelic exchange. Nat Protoc 10: 1820–1841. doi:10.1038/nprot.2015.115.

11. Shanks RMQ, Caiazza NC, Hinsa SM, Toutain CM, O’Toole GA (2006) *Saccharomyces cerevisiae*-based molecular tool kit for manipulation of genes from gram-negative bacteria. Appl Environ Microbiol 72: 5027–5036. doi:10.1128/AEM.00682-06.

12. Bolger AM, Lohse M, Usadel B (2014) Trimmomatic: a flexible trimmer for Illumina sequence data. Bioinformatics 30: 2114–2120. doi:10.1093/bioinformatics/btu170.

13. Dobin A, Davis CA, Schlesinger F, Drenkow J, Zaleski C, et al. (2013) STAR: ultrafast universal RNA-seq aligner. Bioinformatics 29: 15–21. doi:10.1093/bioinformatics/bts635.

14. DeLuca DS, Levin JZ, Sivachenko A, Fennell T, Nazaire M-D, et al. (2012) RNA-SeQC: RNA-seq metrics for quality control and process optimization. Bioinformatics 28: 1530–1532. doi:10.1093/bioinformatics/bts196.

15. Love MI, Huber W, Anders S (2014) Moderated estimation of fold change and dispersion for RNA-seq data with DESeq2. Genome Biol 15: 550. doi:10.1186/s13059-014-0550-8.

16. Huerta-Cepas J, Szklarczyk D, Forslund K, Cook H, Heller D, et al. (2016) eggNOG 4.5: a hierarchical orthology framework with improved functional annotations for eukaryotic, prokaryotic and viral sequences. Nucleic Acids Res 44: D286-93. doi:10.1093/nar/gkv1248.
